# Supplementary material for: Children with disabilities in nutrition programmes: Thematic analysis of training and guidelines
Source: PLOS Glob Public Health. 2026 Apr 15;6(4):e0006188. doi: 10.1371/journal.pgph.0006188 (PMC13082666; doi:10.1371/journal.pgph.0006188)
Supplement: S1 Table — (DOCX) [file pgph.0006188.s001.docx]

| **Type** | **Resource Name** | **Publisher** | **Year** | **Accessible Formats** | **Languages** | **Disabilities** | **Guidance on Local Adaption** |
| --- | --- | --- | --- | --- | --- | --- | --- |
| Training resources | Feeding the Child with Cerebral Palsy | Physiopedia | 2022 | Videos |  | Cerebral Palsy | No |
|  | Resource Library for Identifying Feeding Difficulties in Infants, Screening Children for Feeding Difficulties, and Positioning Children for Mealtimes. | SPOON | 2022 | Illustrations |  |  | No |
|  | Online Course: Introduction to Nutrition and Feeding for Children | SPOON | 2021 | E-learning |  |  | No |
|  | Nutrition integration for the children with special needs: A facilitators guide for Food Security, Nutrition, Livelihoods and Disability Inclusion Project Staffs | LEARN | 2020 | Illustrations, Exercises |  | Cerebral Palsy, Down syndrome, Cleft lip and/or palate | No |
|  | Feeding and Positioning Manual: Guidelines for Working with Babies and Children | Holt International | 2019 | Handouts for Caregivers | Amharic, Chinese, Filipino, Kannada, Luganda, Marathi, Mongolian, Vietnamese, and French (Haitian French) | Autism, Cardiac conditions, cerebral palsy, cleft lip and/or palate, deaf/hard of hearing, down syndrome, fetal alcohol spectrum disorders, prematurity, vision impairments | Yes |
|  | Working with Infants with Feeding Difficulties Training Package | MAITS | 2018 | Videos, Handouts, Exercises, Pictures |  | Prematurity, low muscle tone (Down Syndrome), high muscle tone and cleft/lip palate | Some |
|  | Working with Children with Eating and Drinking Difficulties: A training programme for healthcare specialists in low resource settings | MAITS | 2017 | Videos, Pictures |  | Cerebral Palsy, Autism Spectrum Disorder | Some |
|  | Our Videos: Care of Small Babies | Global health Media | 2017 | Videos | Arabic, Bagla, Bahasa Indonesia, Burmes, French, Ga, German, Hausa, Hindi, Italian, Japanese, Khmer, Kinyarwanda, Kurdish, Luo, Nepali, Persian, Somali, Spanish, Swahili, Turi, Urdu | Prematurity and low birth weight | No |
|  | How to approach Feeding Difficulties in young children | H. R. Yang | 2017 |  |  |  | No |
|  | Assessment of Neurodisability and Malnutrition in Children in Africa | Gladstone et al. | 2014 |  |  |  | Yes |
|  | Growth Charts for Children with Special Health Care Needs | The Centers for Disease Control and Prevention | 2014 | Exercises |  | Down Syndrome, Low birth Weight | No |
|  | Assessment of Growth and Nutrition in Children with Cerebral Palsy | Samson-Fang et Bell | 2013 |  |  | Cerebral Palsy | No |
|  | Feeding problems in infancy and early childhood: Identification and management | Arts-Rodas and Benoit | 1998 | Parent Reporting Sheet for Feeding Assessment |  |  | No |
| Program Package | Responsive Care and Early Learning Addendum | USAID Advancing Nutrition | 2022 | Videos, counselling cards | French, Arabic, Russian, Spanish, Kyrgyz | Congenital Zika | Yes - Comprehensive 5 step approach |
|  | Child Nutrition Program: Community Flipbook | Holt International | 2022 | Picture, Flipbook | Thai, Marathi, Kannada, Chinese, Amharic, Vietnamese, Haitian, Lugandan | Cerebral Palsy, Picky Eating, Stigma, Awareness, Disability-visibility | No |
|  | Ubuntu Manual: Working together with families fo children with developmental disabilities | Ubuntu | 2021 | Videos, Pictures, Exercises | French | Cerebral Palsy | Yes |
|  | Baby Ubuntu | Ubuntu | 2021 | Pictures, Handouts, Exercises |  | Young infants 0-2 years, at risk of Cerebral Palsy | Yes |
|  | Juntos | Ubuntu | 2019 | Photos, Exercises | Portuguese, Spanish, Brazilian Sign Language | Congenital Zika and other Neurodevelopmental disorders | Yes |
|  | Working with Children with Developmental Disabilities and their Caregivers: A Training Programme for Non-Specialists in Low Resource Settings | MAITS | 2018 | Photos, counselling cards, high nutrition recipes | Others available but which unknown | Cerebral Palsy, Down Syndrome, Autism spectrum disorders, Hearing Impairment, Visual Impairment, Epilepsy, ADHD | Yes |
|  | Supporting Families for Nurturing Care: Resource Manuals for Home Visitors | UNICEF, International Step-by-Step Association | 2016 | Videos |  | None specified | Yes |
|  | Prevention Toolkit on Cerebral Palsy | CBM | 2012 | Flipbook |  | Cerebral Palsy | No |
| Program Guidance | Seen, Counted, Included: Using data to shed light on the well-being of children with disabilities | UNICEF | 2022 | **Accessible Formats** | **Languages** | **Disabilities** | **Guidance on Local Adaption** |
|  | Essential Actions on Disability Inclusive Nutrition | UNICEF | 2022 | Videos |  | Cerebral Palsy | No |
|  | Inclusion of Children and Young people with disabilities in routine general healthcare: Practice Guide | UNICEF | 2022 | Illustrations |  |  | No |
|  | Improving Young Children's Diets during the complementary feeding period | UNICEF | 2020 | E-learning |  |  | No |
|  | Disability Inclusive Health Services Toolkit: A resource for health facilities in the Western Pacific Region | WHO Western Pacific Region | 2020 | Illustrations, Exercises |  | Cerebral Palsy, Down syndrome, Cleft lip and/or palate | No |
|  | Monitoring Childrens Development in Primary Care Services: moving from a focus on child deficits to family centered-participatory support | WHO | 2020 | Handouts for Caregivers | Amharic, Chinese, Filipino, Kannada, Luganda, Marathi, Mongolian, Vietnamese, and French (Haitian French) | Autism, Cardiac conditions, cerebral palsy, cleft lip and/or palate, deaf/hard of hearing, down syndrome, fetal alcohol spectrum disorders, prematurity, vision impairments | Yes |
|  | Nurturing Care Handbook, Strategic Action 4: Monitor progress | WHO, World Bank, UNICEF | 2021 | Videos, Handouts, Exercises, Pictures |  | Prematurity, low muscle tone (Down Syndrome), high muscle tone and cleft/lip palate | Some |
|  | Including Children with Disabilities in Humanitarian Action: Nutrition | UNICEF | 2018 | Videos, Pictures |  | Cerebral Palsy, Autism Spectrum Disorder | Some |
|  | A Toolkit for Measuring Early Child Development in Low and Middle-Income Countries | World Bank | 2017 | Videos | Arabic, Bagla, Bahasa Indonesia, Burmes, French, Ga, German, Hausa, Hindi, Italian, Japanese, Khmer, Kinyarwanda, Kurdish, Luo, Nepali, Persian, Somali, Spanish, Swahili, Turi, Urdu | Prematurity and low birth weight | No |
