# Supplementary material for: Children with disabilities in nutrition programmes: Thematic analysis of training and guidelines
Source: PLOS Glob Public Health. 2026 Apr 15;6(4):e0006188. doi: 10.1371/journal.pgph.0006188 (PMC13082666; doi:10.1371/journal.pgph.0006188)
Supplement: S1 File — (DOCX) [file pgph.0006188.s002.docx]

S1_Text: Topic Guide

1. **Main questions**
2. *Prompts*

**Participant Descriptors:**

| Professional Background | O Medicine | O Nutrition / Dietetics | O Program specialist / | O Allied-health professional | O Other |
| --- | --- | --- | --- | --- | --- |
| Type of organisation | O NGO | O Government | O Health | O UN / International Agency | O Other |
| Geographical location (location of global experience) | O UK and Europe | O Asia and the Pacific | O North America | O Sub-Saharan Africa | O Middle-East and North Africa |
| Years of experience post-qualification | O < 5 years | O 5-10 years | O 10-15 years | O 15-20 years | O > 20 years |

Section 1: Introduction/Background

1. **Could you start by telling me a little about your professional background?**
2. *What background or training did you come into the field of nutrition/disability from?*
3. *What led you to have a particular interest in nutrition/disability?*
4. **Where / when did you first notice the relationship between nutrition and disability?**
5. *Why do you think it important to have a focus on disability in the prevention and management of malnutrition?*
6. *Could you describe some of the main challenges children with disability face regarding nutrition?*
7. **How well do you think the link between malnutrition and disability is currently addressed in nutrition (a) guidance and policies (b) programming and (c) training materials ?**
8. *Do you think there are any gaps in the guidelines, and if so, where?*
9. *Who do you think are the key organisations in the field doing disability and nutrition work?*

Section 2: Strengths and Weaknesses of the Guidance, Training and Programmes

1. **What resources have you come across or used regarding disability-specific recommendations for nutrition and feeding?**
2. *Who made the resource?*
3. *What type of resource was it?*
4. *How and where was the resource accessed?*
5. *Who are the intended users of the resource?*
6. *Which themes/specific disabilities are referenced/covered?*
7. **Overall what was your impression of the resource?**
   - 1. What were the biggest strengths and weaknesses of the resources used?
     2. Were there any gaps you identified?
     3. Did the resource align with / reference any other tools or frameworks?

Section 3: Barriers and Opportunities to Implementation

1. ***How and where do you become aware of and share new resources (e.g. professional membership groups, organisation listserv)***
2. ***(If not mentioned) Have you heard of, accessed, or utilised the USAID Feeding and Disability Resource Bank.***
3. **Can you think of any barriers to implementation of these resources?**
   - 1. *How do you think we can improve the dissemination of disability resources?*
4. **What about facilitators?**
   - 1. *Are there any examples of best practice for use of these that you can think of?*

Section 4: Future Development of the Resources

1. **What do you think the next steps are in terms of improving the guidance and programs on disability-inclusive nutrition?**
   - 1. *How well do you think the current recommendations align with work of other sectors: disability, nutrition, health, child development*
2. **What can we do to better standardise how we are supporting these children?**
3. *Would a more standardised resource, that could be referenced / recommended in guidelines, be useful in your work?*
4. *Is there a resource you know about that could be used / adapted, or would a purpose-made new one be better?*
5. *How would you picture this resource looking?*
6. *Would guidance on local adaption of resources be useful?*

**Summary & Conclusions**

Thank you so much for answering all of my questions.

Is there anything you would like to ask or tell me before we conclude the interview?

Now that you have heard the questions, is there anyone else that you would recommend I try to get in contact with for an interview?

Thank you for your time. I really appreciate your contribution to this research project and look forward to sharing the results with you in due course.
