# Supplementary material for: Children with disabilities in nutrition programmes: Thematic analysis of training and guidelines
Source: PLOS Glob Public Health. 2026 Apr 15;6(4):e0006188. doi: 10.1371/journal.pgph.0006188 (PMC13082666; doi:10.1371/journal.pgph.0006188)
Supplement: Attachment — Submitted filename: PGPH-D-25-01205_NHJ.pdf [file pgph.0006188.s003.pdf]

PLOS Global Public Health

Supporting Children with Disabilities in Nutrition Programmes: thematic analysis of training and guidelines key informant interviews

--Manuscript Draft--

**Manuscript Number:**	PGPH-D-25-01205
**Article Type:**	Research Article
**Full Title:**	Supporting Children with Disabilities in Nutrition Programmes: thematic analysis of training and guidelines key informant interviews
**Short Title:**	Nutrition programming for children with disabilities
**Corresponding Author:**	Katie Fulford<br>London School of Hygiene & Tropical Medicine<br>London, UNITED KINGDOM OF GREAT BRITAIN AND NORTHERN IRELAND
**Order of Authors:**	Katie Fulford
	Emily DeLacey
	Fiona Beckerlegge
	Julia Hayes
	Himali de Silva
	Marko Kerac
**Keywords:**	Disability; Nutrition; child nutrition; feeding difficulties; nutrition programmes; malnutrition
**Abstract:**	Children with disabilities are often excluded in nutrition policy, programming and practice. A recently established “Feeding and Disability Resource Bank” collated currently available guidelines and training materials relevant to this group. It forms an important baseline for improved future guidelines and training materials to better support nutrition among children with disabilities. The primary objective of this study was to analyse guidance, existing training materials, programme packages and programmatic guidelines. Semi-structured key-informant interviews were conducted to identify priority areas for improvement of the resources, and to place findings in context with the main barriers and facilitators to their use. Data was analysed deductively to identify themes around the study’s objectives. Guidance included 13 training resources, eight programme packages and nine programmatic guidelines which were mapped for coverage and depth of recommendations for 28 key topics. Key topics included identification of disability, feeding difficulties and malnutrition, management of feeding difficulties and malnutrition and integration of care for a child, family and community. Twelve key informant interviews were conducted. Results indicate that although resources available are growing, few provide a comprehensive approach. Gaps include appropriate assessment of nutritional status (8/30 resources), micronutrient deficiencies (0/30), management of the nutritional quality of a modified diet (6/30) and dehydration (3/30). Resources could be strengthened by filling gaps, standardising recommendations and operationalising how these practices can be integrated into programming. The uptake of these recommendations could be maximized by concurrent consideration of the visibility of disability in nutrition agendas, accessibility and dissemination of the resources and human and resource capacity of the sector. There are some high-quality resources to support children with disabilities, but more are needed to provide comprehensive care. Unless addressed, children with disabilities will continue to experience systemic exclusion from accessing quality care, treatment and prevention of further disability.
**Additional Information:**	
**Question**	**Response**
**Financial Disclosure**	This research received no external funding.

Powered by Editorial Manager® and ProduXion Manager® from Aries Systems Corporation

Enter a financial disclosure statement that describes the sources of funding for the work included in this submission and the role the funder(s) played. This includes grants and any commercial funding of the work or authors.<br><br>This statement will be typeset if the manuscript is accepted for publication.<br><br>*Please review the submission guidelines and the instructions link below for detailed requirements and guidance.*	
**Competing Interests**<br><br>On behalf of all authors, disclose any competing interests that could be perceived to bias this work.<br><br>This statement will be typeset if the manuscript is accepted for publication.<br><br>*Please review the instructions link below and PLOS Global Public Health's competing interests policy to determine what information must be disclosed at submission.*	Emily DeLacey and Julia Hayes work for Holt International and authored one of the guidelines in this review. Himali de Silva has volunteered for health charity MAITS, whose materials were also reviewed.
**Data Availability**<br><br>Before publication, Authors are required to make fully available and without restriction all data underlying their findings. Please see our PLOS Data Policy page for detailed information on this policy.<br><br>A **Data Availability Statement**, detailing where the data can be accessed, is required at first submission. Insert your Data Availability Statement in the box below.	Data is available pending approval from the corresponding author.

Powered by Editorial Manager® and ProduXion Manager® from Aries Systems Corporation

Please see the data reporting section of our submission guidelines for instructions on what you need to include in your Data Availability Statement.<br><br>This statement will be typeset if the manuscript is accepted for publication.<br><br>*PLOS allows rare exemptions to address legal and ethical concerns. If you have legal or ethical restrictions, please use the box below to detail these in full sentences for the Journal team to consider.*	

Powered by Editorial Manager® and ProduXion Manager® from Aries Systems Corporation

Manuscript

Click here to access/download;Manuscript;Full Final Manuscript Disabilities Nutrition.docx

Supporting Children with Disabilities in Nutrition Programmes: thematic analysis of training and guidelines key informant interviews

Short title: Nutrition programming for children with disabilities

Katie Fulford[1,2], Emily DeLacey[2,3], Fiona Beckerlegge[5,], Julia Hayes[3], Himali de Silva[4], Marko Kerac[1,2]

[1]Department of Population Health, Faculty of Epidemiology and Population Health, London School of Hygiene & Tropical Medicine; University of London, Keppel Street, London WC1E 7HT, UK

[2]Centre for Maternal, Adolescent, Reproductive & Child Health (MARCH), London School of Hygiene & Tropical Medicine; University of London, Keppel Street, London WC1E 7HT, UK

[3]Nutrition and Health Services, Holt International; 250 Country Club Rd, Eugene, OR 97401, USA

[4]Evelina London Children's Community Speech and Language Therapy Service, Mary Sheridan Centre for Child Health; 5 Dugard Way, London SE11 4TH

[5] Kyaninga Child Development Centre; Fort Portal, Kabarole District, Uganda

ORCID

Katie Fulford: https://orcid.org/0009-0006-8715-0416

Marko Kerac: https://orcid.org/0000-0002-3745-7317

Fiona Beckerlegge: https://orcid.org/0000-0001-8088-4683

Himali de Silva: https://orcid.org/0009-0000-2640-321X

Julia Hayes: https://orcid.org/0000-0001-5578-5659

Emily DeLacey: https://orcid.org/0000-0003-2286-6430

Conflict of interest statement

Emily DeLacey and Julia Hayes work for Holt International and authored one of the guidelines in this review. Himali de Silva has volunteered for health charity MAITS, whose materials were also reviewed.

Authorship Statement

K.F. and M.K. designed the study. K.F., H.dS., F.B., J.H. E.D. and M.K. contributed to specific areas of the methods, data analysis, statistics, and quality control. K.F. and M.K. had access to and verified the data. K.F. led the data analysis and writing of the first draft of the manuscript. K.F., H.dS., F.B., J.H. E.D. and M.K. contributed to the writing of the manuscript and agreed with the manuscript’s results and conclusions. All authors have read and agreed to the published version of the manuscript.

Funding

This research received no external funding.

Institutional Review Board Statement

This study was conducted in accordance with the Declaration of Helsinki and approved by the London School of Hygiene and Tropical Medicine’s Ethics Committee (ref: 30069).

Informed Consent Statement

Informed consent was obtained from all subjects involved in the study. Written informed consent has been obtained from the participants(s) to publish this paper.

Data Availability Statement

Data is available pending approval from the corresponding author.

Abstract

Children with disabilities are often excluded in nutrition policy, programming and practice. A recently established “Feeding and Disability Resource Bank” collated currently available guidelines and training materials relevant to this group. It forms an important baseline for improved future guidelines and training materials to better support nutrition among children with disabilities. The primary objective of this study was to analyse guidance, existing training materials, programme packages and programmatic guidelines. Semi-structured key-informant interviews were conducted to identify priority areas for improvement of the resources, and to place findings in context with the main barriers and facilitators to their use. Data was analysed deductively to identify themes around the study’s objectives. Guidance included 13 training resources, eight programme packages and nine programmatic guidelines which were mapped for coverage and depth of recommendations for 28 key topics. Key topics included identification of disability, feeding difficulties and malnutrition, management of feeding difficulties and malnutrition and integration of care for a child, family and community. Twelve key informant interviews were conducted. Results indicate that although resources available are growing, few provide a comprehensive approach. Gaps include appropriate assessment of nutritional status (8/30 resources), micronutrient deficiencies (0/30), management of the nutritional quality of a modified diet (6/30) and dehydration (3/30). Resources could be strengthened by filling gaps, standardising recommendations and operationalising how these practices can be integrated into programming. The uptake of these recommendations could be maximized by concurrent consideration of the visibility of disability in nutrition agendas, accessibility and dissemination of the resources and human and resource capacity of the sector. There are some high-quality resources to support children with disabilities, but more are needed to provide comprehensive

care. Unless addressed, children with disabilities will continue to experience systemic exclusion from accessing quality care, treatment and prevention of further disability.

Key Messages

1. Children with disabilities are often excluded from mainstream or widespread nutrition and health services, in part due to practitioners having poor access to high quality nutrition programmes and resources.
2. There are some high quality but under-utilized nutrition program resources available to support children with disabilities, both in specialised programming for children with disabilities and inclusive nutrition programming.
3. More guidance is needed on how to use and integrate inclusive comprehensive services and techniques, while also providing specialised services for children with disabilities.

Introduction

Malnutrition continues to be a significant threat to child health and development globally. UNICEF/WHO/World Bank statistics estimate that some 148 million children younger than 5 years worldwide (22.3%) are stunted, 45 million (6.8%) wasted and 37 million (5.6%) overweight. (1) Millions are also vulnerable to micronutrient deficiencies such as iron deficiency anaemia. (2) Fewer than half of all countries are 'on track' to meet even one of the nine global nutrition targets by 2025. (3) The rate of current progress will result in the 2030 malnutrition targets being missed by more than 39 million children. (1) Some of those most vulnerable are the 240 million children with disabilities, about 1 in every 10 children. (4) (5) (6) A child's likelihood of having a disability by their fifth birthday is now 10 times higher than their likelihood of dying. (7) This reflects both successful child mortality interventions, as well as the neglect of morbidity and prevention in those interventions. Between 1990 and 2016 Sub-Saharan Africa saw a 55% reduction in mortality for children younger than 5 years, and a 71.3% increase in developmental disorders (such as epilepsy, neurodevelopment disability, and autism spectrum disorder). (8)

Malnutrition is well recognized as a major cause of disability at all stages of the life course, particularly during the critical window of the first 1,000 days of life. (9) (10) Maternal and neonatal disorders, where nutrition plays a crucial role, are the leading cause of all disability-adjusted life years (DALYs) (52.47%) and nutritional deficiencies are the third (15.26%). (11) The reverse is also true — children with a disability are at greater risk of malnutrition and consequential ill health. (12) A pooled meta-analysis of data from LMICs showed that children with disabilities had almost three times the odds of being underweight, twice the odds of wasting and twice the odds of stunting compared to children without. (13) (14) Many factors influence the

nutritional status of children with disabilities including presence of feeding difficulties, access to quality care, age-appropriate nutrient-rich foods, feeding practices, environmental factors, resources and societal/cultural norms. (15) Difficulties with feeding can be an early indicator of delayed development or disability risk later in life. Timely identification of these provides practitioners with a golden opportunity to better support families both short and long term.

With malnutrition and disability both being major factors in global health, and clear evidence demonstrating their bi-directional risk relationship, stronger links could produce mutual benefits and improved outcomes for children. (16) The Convention of the Rights of Persons with a Disability and the Convention of the Rights of the Child mandate that all children have the right to accessible and affordable treatment for any disease, illness or health condition. (17) (18)

Many current nutrition programmes fail to adequately consider or support underlying disability. (19) (20) A recent scoping review identified insufficient policies, programmes or evidence to support children with disabilities or feeding difficulties in malnutrition prevention and treatment. (21) Resources that were found during this review were compiled into a publicly available bank. (22) (23) To optimize their future use and value, it is important to understand the details of what these resources and guidance documents currently cover. This knowledge would help professionals supporting children with disability better identify the opportunities for disability-inclusive programming, priority areas for development and strengthening of policy, programming and research.

Aim

To help improve quality of care for children with disabilities in nutrition programmes by evaluating the currently available supporting resources.

Objectives

1. To conduct a thematic analysis of the nutrition resources for children with disabilities.
2. To evaluate whether the resources adequately address the main challenges and needs in programming to support children with disabilities.
3. To evaluate the barriers and facilitators to the use of the current available resources.
4. To generate recommendations for future resource development and programmatic design to strengthen disability-inclusive and disability-specific nutrition.

Materials and Methods

Study Design

This is a mixed methods study using cross sectional data and qualitative key-informant (KI) interviews to explore the currently available resources for supporting children with disabilities in nutrition programmes. We follow the STROBE checklist with secondary additions from the COREQ checklist for qualitative research. This study was conducted in accordance with the Declaration of Helsinki and approved by the London School of Hygiene and Tropical Medicine's Ethics Committee (ref: 30069).

Feeding and Disability Bank Review

We extracted data from a recently compiled bank of resources to create a repository of materials to help nutrition and disability programme managers, government leaders and other stakeholders design and implement effective nutrition programmes for children with disabilities. (21) (22) The bank is now facilitated by UNICEF, although only resources present on the original USAID Feeding and Disability Resource Bank were included in this review. (22) (23)

For inclusion in our study, resources needed to be published in English, targeted to professional audiences, open access and accessible during the search period of June 1 - July 31, 2024. They also needed to include references to both nutrition and disability or feeding difficulties, with any disability or feeding difficulty considered. (21)(22)(23) The resources were only intended for use by family members and resources that focused on professional development of the healthcare worker were excluded from our analysis. Individual screening/assessment tools on the resource bank were excluded from the main thematic analysis but are discussed separately in Table 3.

Key-Informant Interviews

Key-informant (KI) interviews were conducted to complement and provide a contextual backdrop to the results and implications of the thematic analysis. A phenomenological approach was adopted to explore the real world experience of accessing and using these resources. All interviews were conducted by K.F., the principal investigator, with no prior relationships existing between the principal investigator and KIs. A mixture of purposive and snowball sampling was used to identify and invite professionals to share their expertise in a KI interview. KIs were targeted based on their experience at either a programme or policy level, with an extra focus on those with direct service experience. KI included are experts who work in both global nutrition and child disability services and programming. KIs were from a variety of different professional backgrounds, sectors and geographies. Purposive sampling started with four participants, with remaining KIs snowball sampled between 24th June – 16th July 2024.

Information was provided and written consent obtained prior to interviews commencing. The interviews were conducted via Zoom between July 9 and Aug. 7, 2024. Meetings were recorded and transcribed using Zoom's built-in audio transcription software. The transcriptions were checked and edited in Microsoft Word by the interviewer, using audio recording of the meeting. Transcripts were made anonymous through the removal of any identifiable information and categorisation of job roles, projects, locations and organisations.

KI interviews were semi-structured following a topic guide aligned with study objectives (S3). The questions aimed to gain information about the key informant, their experiences, their use of resources, the strengths and weaknesses of the

resources currently available, the barriers and facilitators to their implementation and areas for the development of resources or guidance on nutrition and disability.

Data Collection and Coding

Included resources were categorised as programme packages, programmatic guidance or training manuals. All were reviewed in detail, and topics covered were coded to identify key content areas. Finally, these were grouped into themes. A heat map was used to demonstrate which of the key content areas were identified in each resource and whether any programmatic recommendations were given. This coverage and quality of recommendations followed a key to reduce reviewer bias.

The KI interviews were coded and analysed following a three-step practical thematic analysis approach for multi-disciplinary health research, including reading, coding and theming. (24) Coding was done to identify arising strengths and weaknesses of the resources as well as external facilitators and barriers to their implementation. Codes were then grouped into themes within their brackets of strengths, weaknesses, facilitators and barriers.

Analysis

Interview data on the strengths and weaknesses of various resources were matched and discussed together with the key areas from the thematic analysis: identification, management and integration of care. This combination of results was designed to highlight what the resources and guidance currently cover, and how they meet the needs of professionals supporting children with disability. Presenting key topics in heat maps was designed to help professionals seeking information on a particular topic easily and rapidly to identify the best resources.

Interviews data on the barriers and facilitators to the resource's implementation were analysed and reported separately. This enables identification of opportunities for greater disability-inclusive programming, priority areas for development and strengthening of policy, programming and research.

Results

Of the 86 resources on the Feeding and Disability Resource Bank, 30 met the inclusion criteria for thematic analysis: 13 training resources, eight program packages and nine programmatic guidelines (Figure 1).

Figure 1: Flow Diagram depicting inclusion and exclusion criteria of resources from the Feeding and Disability Resource Bank for thematic analysis.

There were 28 key content areas identified within the literature, under three main themes; identification, management and integration. The first, identification, focused on five key content areas on identification of children with disabilities, feeding difficulties and/or malnutrition. The second included 15 content areas related to managing feeding difficulties and malnutrition, such as recommendations for positioning, feeding equipment, oral support and wider cognitive and socio-emotional development. Finally, the third content area focused on integration of disability services into wider care which comprised eight content areas related to follow-up and a holistic care for children, support for caregivers and addressing environmental barriers such as exclusion, accessibility and stigma. The title, publisher and date are shown in the first heat map table and correspond to the order of numbers in Table 2 and Table 4 with an additional table with the full resource titles in the appendix (S1).

Key Informant Interviews

Of the 14 professionals invited for participation, one KI did not respond and one declined due to a perceived lack of specific knowledge on the subject *(Table 1)*. Twelve interviews were conducted.

Table 1: Description of Key Informants

**Description of 12 Key Informants**	**Number % (percentage)**
**Sex**	
Female	11 (92)
Male	1 (8)
**Professional Background**	
Medicine	2 (17)
Nutrition/Dietetics	5 (41)

Programme Specialist/Child Development	2 (17)
Allied-health sectors (Speech and language and Physiotherapy)	3 (25)
**Professional Sector**	
Health Sector	3 (25)
Non-Governmental Organisation	6 (50)
Governmental Organisation	1 (8)
UN or Other International Agency	2 (17)
**Years of Experience Post-Qualification**	
<10	3 (25)
10-20	3 (25)
>20	6 (50)
**Geographical Location**	
Sub-Saharan Africa	3 (25)
Europe	3 (25)
North America	6 (50)
**Number of Participants with Experience in Each Global Region**	
Asia and the Pacific	7 (58)
Middle East and North Africa	2 (17)
Sub-Saharan Africa	10 (83)
Europe	5 (41)
North America	7 (58)

Disability Identification

Table 2: Heat map depicts coverage and recommendations of key content areas under the themes of identification of disability, feeding difficulties and malnutrition, within the resources from the Feeding and Disability Resource Bank (22). Red indicates not acknowledged, light blue: acknowledged but no guidance, medium blue: acknowledged and some guidance (1-2 recommendations) and dark blue: acknowledged and comprehensive guidance (>3 recommendations).

Type	Resource Name	Publisher	Year	Assess ment tool for	Develo pment al	Assess ment of	Clinical Signs of	Assess ment of
TM	Feeding in Children with Cerebral Palsy (25)	Physiopedia	2022					
	Resource Library (26)	SPOON	2022					
	Introduction to Nutrition and Feeding for Children (27)	SPOON	2021					
	Nutrition integration for the children with special needs (28)	LEARN	2020					
	Feeding and Positioning Manual: Guidelines for Working with Babies and Children (29)	Holt International	2019					
	Working with Infants with Feeding Difficulties (30)	MAITS	2018					
	Working with Children with Eating and Drinking Difficulties (31)	MAITS	2017					
	Our Videos: Care of Small Babies (32)	GHM	2017					
	How to approach Feeding Difficulties in young children (33)	H. R. Yang	2017					

	Assessment of Neurodisability and Malnutrition in Children in Africa (34)	Gladstone	2014					
	Growth Charts for Children with Special Health Care Needs (35)	CDC	2014					
	Assessment of Growth and Nutrition in Children with Cerebral Palsy (36)	Samson-Fang	2013					
	Feeding problems in infancy and early childhood (37)	Arts-Rodas	1998					
PP	Responsive Care and Early Learning Addendum (38)	USAID	2022					
	Child Nutrition Program: Community Flipbook (39)	Holt International	2022					
	Ubuntu Manual (40)	Ubuntu	2021					
	Baby Ubuntu (41)	Ubuntu	2021					
	Juntos (42)	Ubuntu	2019					
	Working with Children with Developmental Disabilities and Caregivers (43)	MAITS	2018					
	Supporting Families for Nurturing Care (44)	UNICEF	2016					
	Prevention Toolkit on Cerebral Palsy (45)	CBM	2012					
PG	Seen, Counted, Included (5)	UNICEF	2022					
	Essential Actions on Disability Inclusive Nutrition (46)	UNICEF	2022					

Inclusion of Children and Young people with disabilities in routine general healthcare (47)	UNICEF	2022					
Improving Young Children's Diets during complementary feeding (48)	UNICEF	2020					
Disability Inclusive Health Services Toolkit (49)	WHO	2020					
Monitoring Childrens Development in Primary Care Services (50)	WHO	2020					
Nurturing Care Handbook, Strategic Action 4 (51)	WHO	2021					
Including Children with Disabilities in Humanitarian Action: Nutrition (52)	UNICEF	2018					
A Toolkit for Measuring Early Child Development in LMICs (53)	WB	2017					

** TM: Training Manual, PP: Programme Package, PG: Programmatic Guidance

Identifying disability is paramount to the appropriate management of nutrition and health. Developmental monitoring is one method that can be used to screen children for early signs of disability. Of the 30 resources, 12(40%) reference a standardized assessment tool for monitoring developmental and disability (Table 3). These are mostly in programmatic guidance, as only two are training manuals and two program packages. Training manuals and program packages more commonly contain information on the developmental milestones (40%), which equip health workers to clinically assess evidence of age-appropriate development. Guidance on how development milestones relate to feeding skills, such as use of the tongue to move food to the back of the mouth for swallowing or ability to hold feeding utensils, was only found in five of the resources (17%). In low-resource settings, standardized assessment tools can aid less-experienced or semi-skilled health workers to appropriately identify children with disability or developmental delay and manage accordingly, particularly due to the heterogeneity of how different disabilities present and children's individual functional abilities.

"We were just having these re-admissions, and it meant in terms of disability they were getting further and further behind their developmental milestones because we were not addressing the root cause of the malnutrition [… that the child had a disability]." Participant 3

"We're relying on professional knowledge and skills and clinical reasoning to be able to adapt accordingly. That's what makes it very difficult when you've lower-level health workers or semi-skilled health workers who don't have that." Participant 2

The most referenced assessment tools are the Washington Group Short Set on Functioning and UNICEF's Module on Child Functioning Questionnaires (54)(55). Few recommendations for developmental monitoring are specific to the context of

nutrition programming. Resources, such as the World Bank Toolkit for Measuring Early Child Development in Low- and Middle-Income Countries and Monitoring Children's Development in Primary Care (53) (50), indicate nutrition services as an opportunity for developmental monitoring but give no specific recommendations how to integrate the practice into that context. The Washington Group Questions are for children aged 5 years and older and the Child Functioning Module for 2-4 years and 5-17 years (54) (55). In the context of a nutrition programme, these assessment tools are relevant only outside of the first 1,000 days of life, where interventions to support development, nutrition and health are known to have the greatest effect and long-term impact.

"Under 6 months it's difficult to diagnose [developmental delay and disability] as they're not going to fit the screening tools. This is why there is this focus on feeding difficulties rather than outright disability. Moving it so it is less stigmatized, so if the mother is saying 'my infant takes a long time to feed', there are things we could do then." Participant 4

Table 3: Developmental Screening and Feeding Assessment Tools in the Feeding and Disability Resource Bank

	**Assessment Tool**	**Publisher**	**Year**	**Development**	**Disability**	**Feeding**
1	Malawi Development Assessment Tool: IDEC Monitoring Tool Version 1.3 (56)	University of Liverpool	2020	x		
2	Washington Group Short Set on Functioning (WG-SS) (54)	Washington Group-on Disability Statistics	2020	x		
3	Module on Child Functioning Questionnaires (55)	UNICEF and Washington Group	2016	x		

4	Modified Checklist for Autism in Toddlers, revised, with follow-up (M-CHAT-RF) (57)	D. Robins	2018		x	
5	Infant and Child Feeding Questionnaire (58)	Feeding Matters	2020			X
6	Eating and Drinking Ability Classification System (EDACS) (59)	Sussex Community NHS Foundation Trust	2013			X
7	International Dysphagia Diet Standardisation Initiative Framework (60)	IDDSI	2019			X

Identification of Feeding Difficulties

Only 5/30 (17%) resources describe how to conduct a feeding assessment and none refer to the standardized feeding assessment tools currently accessible in the resource bank: the Eating and Drinking Classification System for Children with Cerebral Palsy (EDACs) and the Infant and Child Feeding Questionnaire (Table 3). The resource bank also contained a classification system of modified diets for the management of dysphagia, the IDDSI Framework, which is similarly not referenced in any of the other resources but currently a standard tool used in the field of nutrition. Guidance on identifying clinical signs of feeding difficulties are included in nine of the training resources (70%) and five programs (63%).

Monitoring nutritional status and growth

Recommendations for monitoring nutritional status and growth, specific to children with disability, were only described in 8/30 resources (27%). None of the program packages give recommendations on identifying malnutrition or measuring nutritional status.

*"Classically malnutrition is still [*identified*] through anthropometry, and you can't measure weight and height on a lot of these children, depending on what the disability is. MUAC will be good for some, but not for others." Participant 4*

As MUAC, weight and height are standard global measurement indicators, many guidelines such as the WHO 2023 Wasting Guidelines or Sphere standards use them for screening, monitoring and eligibility criteria for treatment. (61) (62) Consequently, many children with disabilities are excluded from appropriate assessment and therefore stopped from receiving treatment and support.

"A lot of children won't be picked up by malnutrition programs because they say 'they have a disability, there's no guidelines to screen these children and we don't know how to include them, track their progress, determine if they're improving or not improving.'" Participant 5

The CDC Growth Charts for Children with Special Healthcare Needs contains the most comprehensive guidance for adapting anthropometry practices. (35) It describes how the most important practices in growth monitoring are not limited to which measurement is taken but the consistency, accuracy in technique and repetition of measurement over time. The guidance includes alternative measurement options such as crown-to-rump length recumbent or sitting, arm-span or segmental lengths such as upper-arm and lower-leg length which can also be plotted on growth charts to monitor a pattern. Tracking the indicators that you can measure, and having a formal approach to recording those measurements provide a reference point to monitor an individual child. Wider guidance could reiterate this and provide caveats for how to identify, admit and monitor children in programs when anthropometry is not possible.

"Even though there's no internationally agreed global guidance on it, you can at least track children month to month, and they should not be worsening from where they started. If they can't extend their legs, we're not taking a length measurement, but will still get weight, head circumference, MUAC." Participant 5

Recommendations in the training manuals and guidelines were only for anthropometry and growth monitoring; there was no mention of micronutrient deficiencies or clinical signs of malnutrition.

Management

Table 4: Heat map demonstrating key content areas under the theme of Management of Feeding and Malnutrition, in the resources from the Feeding and Disability Resource Bank. (22) Red indicates not acknowledged, light blue: acknowledged but no guidance, medium blue: acknowledged and some guidance (1-2 recommendations) and dark blue: acknowledged and comprehensive guidance (>3 recommendations).

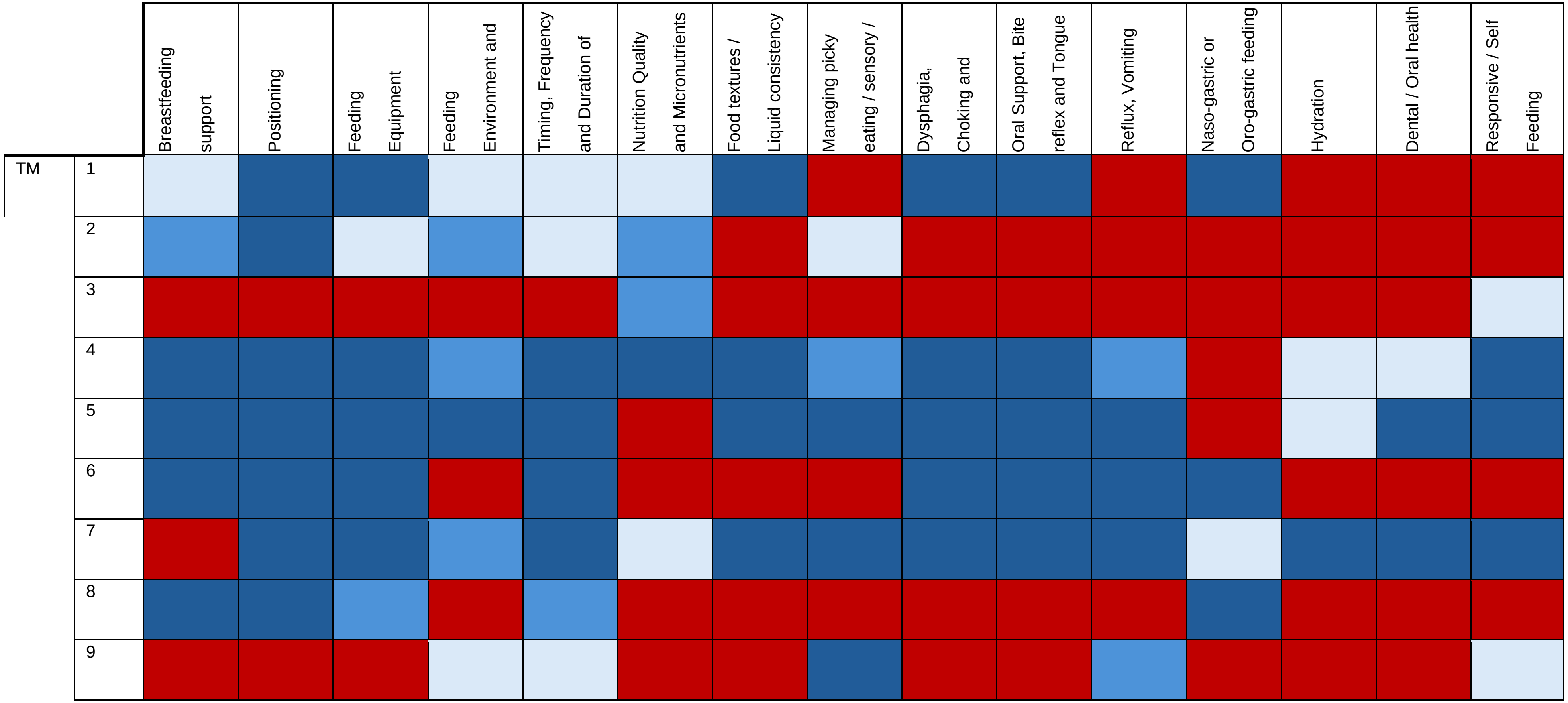

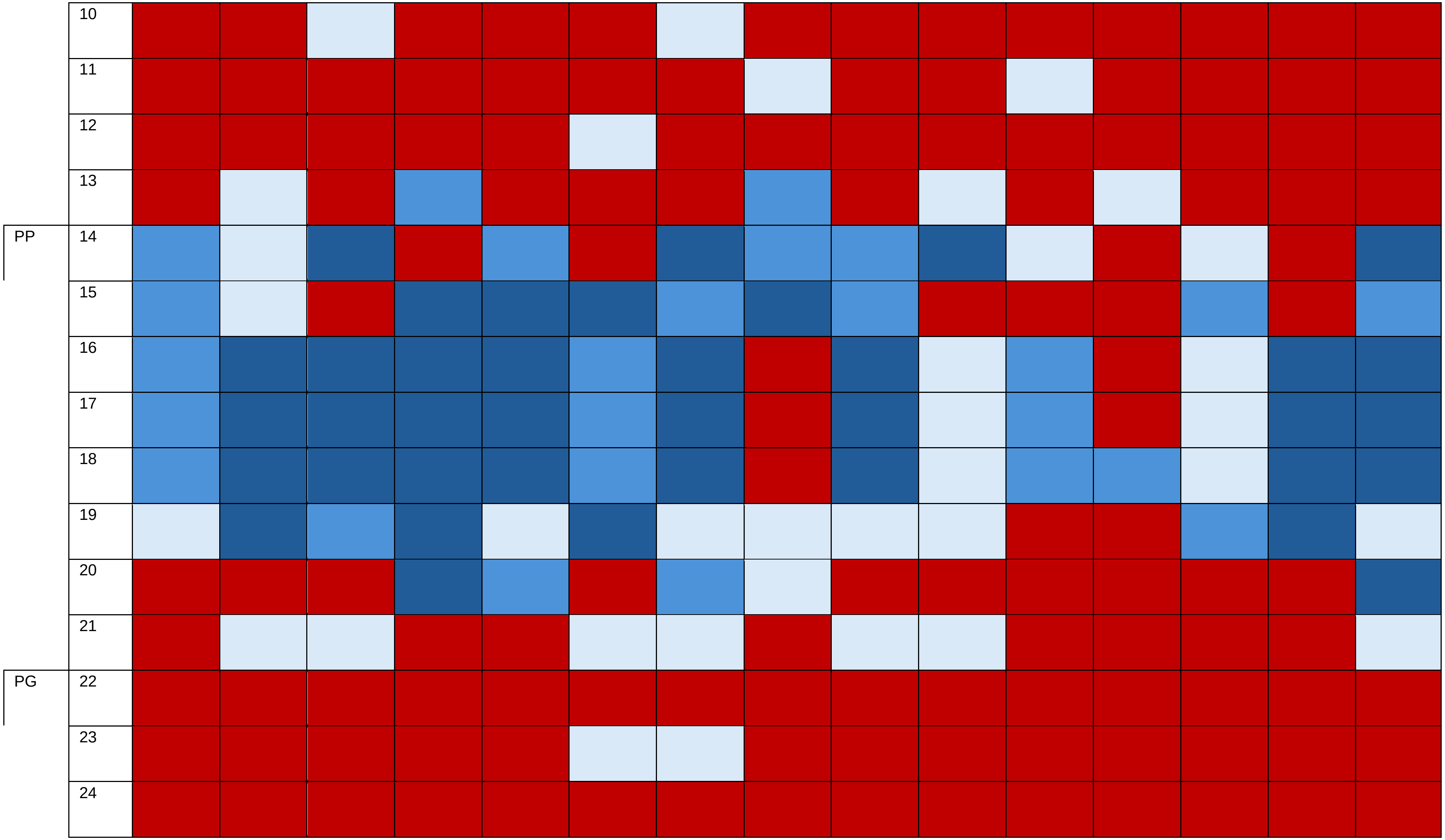
10
11
12
13
PP
14
15
16
17
18
19
20
21
PG
22
23
24

25															
26															
27															
28															
29															
30															

* TM: Training Manuals, PP: Programme Packages, PG: Programmatic Guidance

Table 4 shows recommendations covering the different aspects of management were varied and sparse across the resources except for a few key comprehensive documents. Resources covered many different factors around supporting nutrition for children with different disabilities and clinical manifestations, 21/30 (70%) resources provide recommendations for at least one aspect of clinical care but only 9/30 (30%) cover at least half of the topics. Recommendations for managing feeding are largely absent from the guidelines. UNICEF's guidance on Including Children with Disabilities in Humanitarian Action: Nutrition is an exception and demonstrates how disability-specific recommendations can be integrated into guidelines. The most comprehensive resources are from Holt International, LEARN, Ubuntu and MAITS, which were intentionally designed to improve skills related to feeding and nutrition to support children with disabilities. The most common recommendations were around positioning, timing, frequency and duration of feeding, feeding environment and hygiene, and feeding equipment each found in 12 different resources.

"The risk of aspiration, and chronic upper respiratory infections … these things can start pushing children into that cyclical interaction with malnutrition, disability and infection." Participant 5

There are also some topics and populations not covered as comprehensively in the resources, including nutrition quality of diets, sensory challenges in feeding, hydration, reflux, vomiting and wider medical support. KIs highlighted that the quality of the diet is a priority area for all nutrition programming but has particular importance for children on modified diets, and children whose diet may be very restricted due to food aversion, picky eating and sensory sensitivities to food but resources on the modification of food or managing restricted eating rarely include consideration of the nutritional quality of the diet.

“We find children with disabilities end up being bottle fed for a long time because they think this is the only thing they’re used to, it’s the only thing they’ll tolerate. They’re getting rice porridge in a bottle for the next 10 years, which is not nutritionally adequate.” Participant 5

Key informants discussed how recommendations for a nutritionally balanced and modified diet will look different in different contexts depending on access to and acceptability of varying ingredients and cooking methods, particularly for thickening foods and recommendations need to consider the quality and diversity of macro- and micro-nutrients.

“Up to 65% of children generally in [place redacted] are anaemic, so you wonder what percentage of children with disabilities, who have an even worse diet, would be, and how much does that restrict their improvements." Participant 2

The MAITS training program on developmental disabilities for non-specialists gives guidance on nutrition quality and even contains recipe cards for highly nutritious meals of different textures and consistencies. (43) There is increasing recognition of developmental difficulties and sensory challenges with food, textures and taste. KIs highlighted that the resources needed to also recognize these issues with clinical recommendations for identification and management. Guidance for newborns and young infants with disabilities who are breastfeeding or bottle feeding was also limited. More medical topics, including reflux and vomiting (8/30 resources, 27%), oral support and reflexes (6/30, 20%), dental and oral health (6/30, 20%) and hydration (3/30, 10%) are covered limited in the resources. Hydration was highlighted as a key area of importance by KI, but was poorly covered in the resource documents with only one giving recommendations to consider and monitor hydration status. There were also limited resources on reflux and vomiting for

children with low muscle tone or poor positioning. KI mentioned how clinicians providing direct services are often nervous about implementing feeding practices.

Integration of Care

Table 5: Heat map demonstrating key topics related to integration of care. Red indicates not acknowledged, light blue: acknowledged but no guidance, medium blue: acknowledged and some guidance (1-2 recommendations) and dark blue: acknowledged and comprehensive guidance (>3 recommendations).

	Multi- disciplinary manageme	Social and Emotional Developme	Play, learning and	Maternal and Family psychosoci	Supporting the Family	Accessibilit y	Awareness and Rights	Addressing Stigma and Discriminati
1								
2								
3								
4								
5								
6								
7								
8								

9								
10								
11								
12								
13								
14								
15								
16								
17								
18								
19								
20								

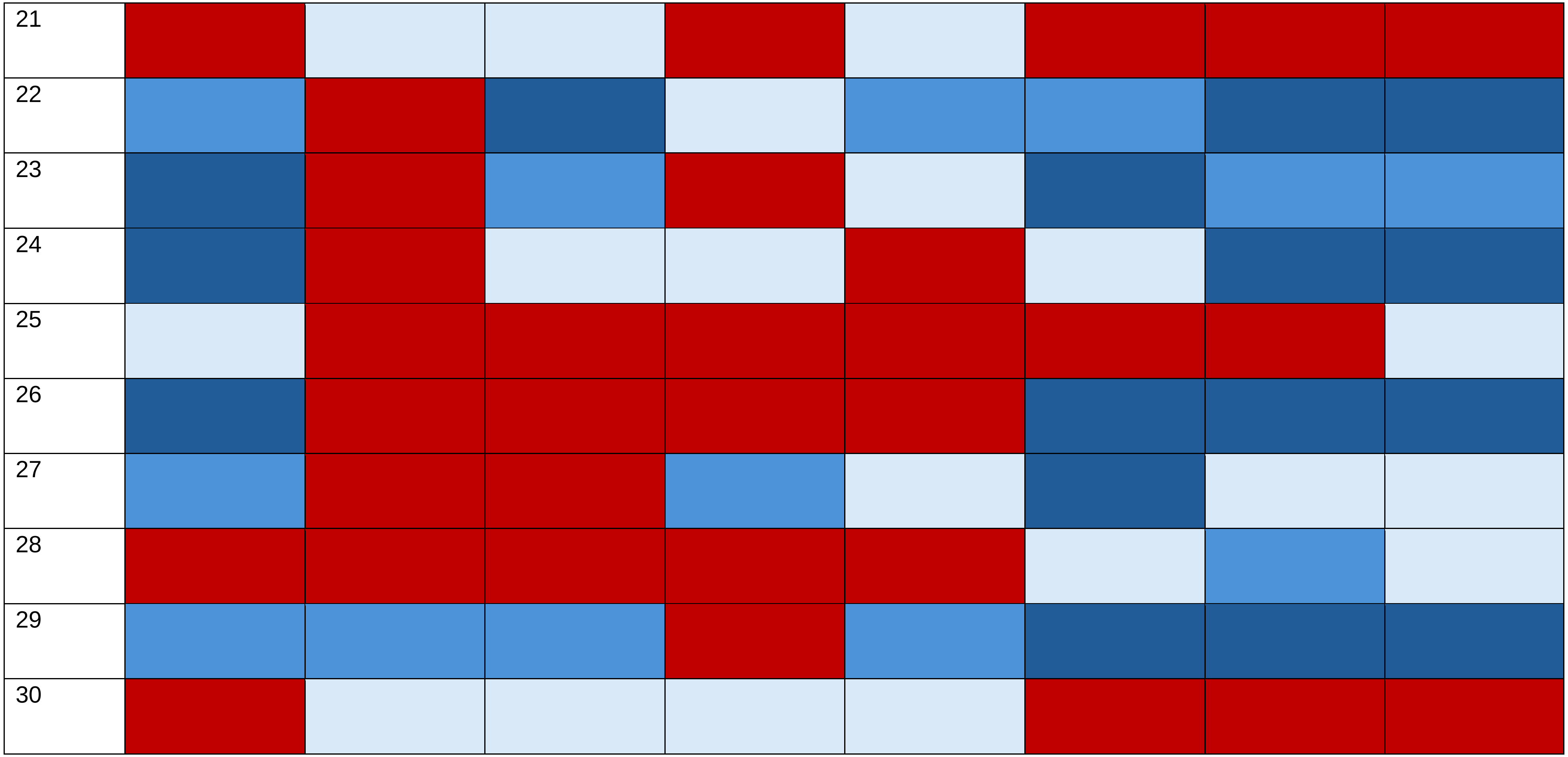
21
22
23
24
25
26
27
28
29
30

In Table 5, 19/30 (63%) of the resources identify the need for a multi-disciplinary approach to care for children with disabilities but few contain examples of what exactly to include. Examples of holistic care found in the thematic analysis include the emotional and cognitive development of the child, social and psychosocial support for the family and caregiver, and broader recommendations to address barriers in the community such as accessibility to services, stigma and discrimination. Recommendations for at least one of these aspects are given in 4/13 (31%) training manuals, 6/8 (75%) program packages and 8/9 (89%) guidelines. Only 9/30 (30%) resources include recommendations to address more than three aspects of wider care. The Nurturing Care Framework, the Ubuntu Programmes and UNICEF's Including Children with Disabilities in Humanitarian Action include recommendations for six or seven different aspects of holistic support. (40–42,44,52)

Support for the Child

Key informants reported that children with disabilities are less likely to access other health services or be reached by programmes in places such as schools. These children are more likely to have comorbidities increasing their risk of malnutrition, and poor response to treatment and support. One described the challenges experienced with malnutrition and how those affected treatment and rehabilitation in physiotherapy.

"A lot of the referrals that we were getting were children who needed corrective and acute management of malnutrition before we could start any rehabilitation process because any energy they were using we had to preserve for survival." Participant 2

Some of the programs connect feeding with support for the child's cognitive, social and emotional development. However, these recommendations are less common in

the training resources and programmatic guidance and therefore KIs identified that unless those specific programs are being implemented, the child's socio-emotional and cognitive development goes unconsidered.

Support for the Family/Caregivers

One common theme from interviews was the impact of disability on the child and their family or caregiver however resources with information for practitioners to support caregivers were limited. Only one of the training resources (8%) and two program guidelines (22%) give any recommendations to support the families' needs, which was incongruent with KI descriptions of the necessity of kitchen gardens, cooking demonstrations and finance programs.

"Most of them come from socially disadvantaged backgrounds, the risk is heightened for children with disabilities. If we look at the mothers, they are also malnourished and they are also at a higher risk of giving birth to a child with a cleft lip and palate." Participant 1

KIs felt this relationship with poverty is not addressed adequately in programmes, training or guidelines.

"Broader support to the family is so important. Thinking about if this child comes to me, do I have a way to check that they are at least connected to someone who can help them navigate whether or not there's social protection programs they can enroll into." Participant 10

Three resources (10%) gave recommendations for a caregiver's emotional and psychosocial health or wellbeing. There were also three other resources on caregiver psychosocial support in the resource bank, but these were excluded from

in-depth analysis in this paper due to a lack of any reference to the relationship to disability or nutrition.

Context

Two resources were developed for children in specific contexts: emergency and humanitarian needs. KI indicated that the overarching guidance in many resources were useful in a wide variety of settings, but they also spoke of the number of contextual gaps missing from the literature. KI highlighted the value of contextually adapted recommendations for improving the specific care and support needed for individual children.

"The institutional care setting is often overlooked [and many children with disabilities live in care], and it is such a prime example of nutrition affecting disability, disability affecting nutrition, all affecting caregiving. It's the whole cycle of it all. Resources for that context are severely lacking." Participant 7

Only 2/30 (6%) resources mentioned children in institutional care.

Stigma and Discrimination

Key informants highlighted the barriers to children accessing appropriate nutrition support as a key issue. Five of the resources discuss wider environmental and community factors that prevent children from accessing care, including stigma and discrimination. Recommendations or resources for addressing societal stigma and supporting children's rights are limited in training manuals.

"We have to consider that in a lot of these communities, there's a ton of stigma around disability and misperceptions of what causes disability. A lot of our health

workers come from those same communities. A lot of times, the health workers hold those same perceptions and blame mothers" Participant 10

"How much is because of the feeding difficulty related to their disability, and how much of it is stigma, how much of it is poverty, what is the root cause of these children's malnutrition? Our gut feeling is it's about 30% feeding difficulties related to the disability and the rest is more poverty or stigma." Participant 2

Figure 2: Suggestions from Key-Informants on How to Strengthen the Resources

509

Recommendations on future development of resources

Recommendations from KI focused on the need for standardized resources and recommendations across the three areas:

- identification of disabilities,
- management of disabilities and malnutrition, and
- integration of wider care considerations for children with disabilities.

Within each of these three sections, gaps in resources, the need for standardized recommendations and guidance on how to operationalize were highlighted as key points of issue. KIs described the challenge for direct service providers in using the many currently existing resources to achieve a comprehensive needs assessment and management strategy, highlighting the need to use several resources. A common theme included standardising the existing recommendations and resources, for example having comprehensive manuals for disability-specific nutrition training and simplified recommendations that could be used to integrate disability into broader guidelines. KIs suggested that the most important topics to include are tools for screening development and disability, red flags for feeding difficulties (during breastfeeding, bottle-feeding and after transitioning to solid foods) and first-line management strategies of positioning and texture and consistency modification for a nutritious diet. Commonly suggested recommendations included tools to direct professionals to specific resources or tools they need. Integration of disability services into existing programming was also identified as a key priority area, especially for breastfeeding programming or complementary feeding programming which are also necessary for children with disabilities. KIs discussed needs for guidance on how to improve existing nutrition programmes to be disability-inclusive

or when is best to have standalone disability programs or how to integrate disability recommendations into programming.

"We are missing more operational guidance. What does it look like to be inclusive? What is it that we really want to see happen in those programmes? We hear it from our country offices 'what is it exactly that you're asking us to do?'" Participant 10

One example given suggested recommendations for multi-disciplinary care should be accompanied by guidance on what exactly to include, in what settings and how to develop the expertise or referral pathways so that practitioners know what to expect when implementing the programs. KIs acknowledged that hesitation to establish formal guidance was due to weak evidence on these issues.

"We don't have great data informing, 'If you do this, you're going to have these results,' but we certainly know that if you do nothing, you're going to have these really bad results. We know some things we can be doing and we need to understand how to do them better." Participant 10

The more disability is integrated into nutrition programming, the more opportunity it provides for implementation research. Some program packages we reviewed were particularly strong, especially those by Ubuntu. Thus, instead of establishing entirely new programmes, implementation research could help refine and standardize existing ones.

"More implementation research to understand those pieces so that the tools themselves can be strengthened and streamlined to what really works best." Participant 10

Identified Barriers and Facilitators to Implementation

KI highlighted that there are external factors both promoting and inhibiting guidelines uptake and implementation. These barriers and facilitators include the visibility of disability in nutrition agendas, access to the relevant resources and the capacity of the system to adopt the recommendations. These priority topics must be addressed for improved future practice (Figure 3).

Figure 3: Recommendations for removing barriers to the implementation of disability and nutrition resources across the themes of visibility, access and capacity

Accessibility

KIs discussed improving access as a key way to improve implementation including improving accessibility to the resource literature (i.e. physical access, printed copies), language accessibility (i.e. translating manuals into more languages/ translation costs). Contextualization is also needed, adapting resources to local contexts, languages and geographies. Not having a central location for resources and having to search many locations (i.e. newsletter, journals, webinars, websites) was identified as a key barrier to accessibility. The Feeding and Disability Bank was highlighted as addressing this problem well as it provides a centralised platform for resources to be more accessible. Even then however, access to some of the Feeding and Disability Bank resources was limited (i.e. only accessible by entering personal details, through organisations' own internal pages or behind paywalls). Eight resources from the bank were excluded from this thematic analysis because they were not open access. KIs reflected that even though electronic access is improving, there remains accessibility issues for many countries, limiting dissemination and utilization of resources. Many of the larger program packages and training manuals are now available in different languages, but translated versions are

limited, mainly due to translation costs (S2). Existing translations may also be poor or lack contextualization.

“The interpreters that we use at [organisation redacted] for all of our training and for the actual translation of the manual, have been outstanding. They will even give us feedback in real time like, ‘That's not going to make sense to them or maybe we should try to say in a different way, because that might come across differently.’” Participant 12

The applicability of recommendations within the local context is also important. One KI described how developmental milestones for feeding skills can differ depending on the child’s environment and prevailing cultural norms. Many training manuals and programs also now include recommendations and guidance on local adaption (S2). Also, the strength of personal and professional networks for sharing resources was a strong theme throughout the interviews. Informal communication networks, such as WhatsApp were described as one method for sharing existing resources, advice and support. First-hand experiences from the KI‘s networks were also mentioned as particularly helpful:

“Using other people’s experiences on the ground, ‘How does this work in this setting?’, is definitely more helpful than guidelines that then have to be adapted to disability, or to this setting.” Participant 2

Several KIs mentioned using online discussion platforms, working groups or listservs as useful methods for resource sharing. These supported building networks and sharing case-studies and advice, advocacy and identification of future priorities for the field.

Capacity

Despite resources being visible and accessible on the bank, barriers were also identified in workforce capacity, understanding and ability to implement recommendations within routine clinical practice.

"There's a big gap in practical training for staff. Time and time again, that's the feedback we've had that they just don't know how to apply it." Participant 8

Many of the resources include training programs for health workers, or have supplementary pictures, videos and even participatory learning activities to help improve understanding of the recommendations (S2). However, more focus on disability/nutrition interactions are needed right from basic/undergraduate training through to post-qualification/postgraduate training. This would ensure that staff have not only theoretical understanding but also the practical knowledge and expertise to use the strategies in everyday clinical practice. Improvements in integrated curricula on care for children with disabilities was discussed as impacting later use of resources, as many current courses do not include any training on disability.

"Disability is there, like it or not, it's part of us. To make it something you learn after you qualify, that's not good." Participant 8

Other limitations reported to use of resources included: time, competing demands from other duties, funding and capacity of practitioners/staff:

"It's really important to think about who we are addressing when we are producing these manuals. The same nurses who have to do the bloods, the weights, are now expected to also help with the feeding. It can't become a part of people's existing job because it just doesn't get done." Participant 8

Multi-track approaches were considered essential but hard to manage for staff with limited time or knowledge, such as providing direct feeding therapy for children and providing feeding training for caregivers. Some of the resources are written so that all individuals, regardless of clinical background, could implement the practices.

Visibility

Both thematic analysis and interviews show that international commitment to disability is growing in the field of nutrition, especially with the creation of guidance from UNICEF's Disability Inclusion Policy and Strategy. (63) However, interviews also revealed that one of the limitations of a resource bank is that it only targets one side of a twin-track approach to disability inclusion; tailoring disability-specific programs, without also ensuring integration into mainstream programming and guidance.

"Where it is now, it is those who are interested, and those who are struggling with this group of children, who are looking for these resources and trying to apply them, but it's not systemic." Participant 10

"[The International Milk Code], that's a big one, talking about breastfeeding and breast milk substitutes and there's no mention of children with disabilities and what their needs might be." Participant 5

KIs also discuss the lack of data collection for children with disabilities, limiting inclusion and development of resources or evaluation of programmes/practices. They also raised the need for more nuanced indicators for children with disability to reduce the risk of those children being excluded from programmes and to capture the impact of treatment on functioning and quality of life (i.e. quality of life indicators may improve far more than anthropometric indicators).

"Nutritional support might be changing quality of life and ease of care." Participant 4

Based on coverage of key content and depth of recommendations in the heat maps, these resources were identified to be particularly strong and suitable for priority use in the short-term.

- MAITS and Holt’s manuals for training professionals on disability-specific feeding and nutrition
- Ubuntu programmes for educating caregivers on appropriate feeding and development
- UNICEF’s guideline, Including Children with Disabilities in Humanitarian Action: Nutrition, for strengthening disability inclusion in mainstream nutrition programming.

Discussion

The growing number of resources available, and the variety of topics covered, demonstrates increasing awareness of disability in the field of child nutrition. However, few resources give a comprehensive account of how to identify children at risk, how to appropriately manage their feeding and nutrition and how to integrate that within a broader approach to care. Major gaps exist in screening and assessment tools, recommendations on the nutrition quality of a modified diet and associated medical risks such as micronutrient deficiencies, dehydration and reflux. Health workers can thus be left ill-equipped to manage these challenges and children with disability risk being systematically excluded from or poorly managed in nutrition programmes.

Suggestions to strengthen the resources, and consequently support better care, include improving, standardising and operationalising the guidance and increasing both number and quality of available resources. This should include recommendations, resources and programmatic guidelines for disability-specific programming, as well as prioritising disability-inclusion in wider guidance.

Implementation of current resources and recommendations is limited by workforce capacity, resource accessibility and visibility of disability across nutrition agendas. Therefore, effort is needed to remove these barriers via strengthening training pathways, allocating appropriate funding and continuing advocacy.

The paucity of recommendations on disability in nutrition has been documented in other work. (20) (64) (16) A guideline review of more than 70 international, national and local nutrition protocols found only three with a section dedicated to disability. (65) The main takeaways from that review, and the accompanying qualitative interviews, included similar recommendations to those of this analysis — the

importance of active screening for disability in malnutrition treatment programs, the need for guidance on providing multidisciplinary management for a child and family, and alternative indicators that can be integrated into nutrition coverage surveys, monitoring and evaluation. The report, ”Seen, Counted, Included” reinforced the importance of improving assessment and data: One in four children with disability had missing height measurements and one in five missing their weight measurements, compared to 1 in 20 and 1 in 30 respectively for children without disability. (5)

Other literature reinforces the value of standardized assessment and screening tools for identifying those needing wider supportive care. For example, using Washington Group Questionnaires in a cohort of malnutrition survivors in Malawi, identified many physical and behavioral difficulties which previous clinical assessment had missed. (14) The feasibility of integrating developmental and disability screening into existing child health and nutrition programmes has been demonstrated in a systematic review. (66) Other literature suggests that having disability or feeding screening and assessment for younger infants, such as through universal newborn screening, can increase the identification of congenital disorders and delays, thereby improving early intervention and preventing poor health and nutrition outcomes. (67)

A scoping review, done at the time of establishing the Feeding and Disability Resource Bank, identified similar barriers to the implementation of the resource literature including poor knowledge among health and nutrition professionals on identification of feeding difficulties and how they relate to disabilities. (21) This lack of knowledge was also found to be driven by inadequate training for staff, insufficient funding to improve training and inadequate standardized policies, programmes and evidence to follow.

Strengths and Limitations of the Study

This project builds on previous work done in compiling the resource bank, offering detailed information on exactly which topics and issues are covered in the resources, including neglected topics such as hydration. It also offers valuable user perspectives on those resources via our KI interviews. We acknowledge that other relevant resources may exist outside of the resource bank, have been added since we did this review, or the literature is evolving and documents may have had minor changes. We were also unable to review some resources due to them not being open source (e.g. SPOON’s feeding and nutrition package for vulnerable children, highlighted as useful by one of our KIs). Mixed methods improved the rigour and value of our study, placing results of the guideline review in context. We took steps to bias by having a wide KI interview base and conducting interviews to a point of saturation. We do, however, acknowledge limitations due to small sample size, relying on snowball sampling and having only one coder. The key-informants chosen varied in background but were English-speaking and mid- to full-career and may not represent all perspectives of individuals working in the field of nutrition and child disability.

Recommendations

One priority research area is implementation research of existing resources and programmes to help refine and adapt recommendations in different contexts. This includes local validation of the assessment and screening tools to improve the identification of children in programmes. Similarly, research is needed on appropriate anthropometric and outcome indicators that can be used to identify and monitor children with disability. Having these indicators would also enable researchers to

capture the scope of disability in programmes as an advocacy tool to better direct allocation of resources and funding.

Considering the high care demands on health care systems, communities and caregivers and the need for community capacity-strengthening that arose in this report, an important follow-up would be a similar analysis of the resources intended for use by the community-specific programs or by caregivers.
Understanding/expanding the adaptability of key community resources, such as MAITS' Guide for Parents: Caring for a Child with Developmental Disabilities and Holt's Community Resource Manual, would strengthen the overall picture of the available literature. (68) (69) Current practice should use these results to identify resources most suitable to individual contexts or prioritise high-quality appropriate resources.

Other research could build on this analysis by using an evaluation tool, such as the AGREE checklist, to determine the standard of the existing resources and clinical guidelines. (70)

Although there are still gaps, this project highlights that existing recommendations and management options could already be doing more to improve disability inclusion and support. Integrating these resources into policy could shift the dynamic and enable a more proactive approach to earlier identification of underlying disability and improved care for children.

Conclusion

There are few training materials, program packages or guidelines providing comprehensive recommendations specific to the nutritional management of children with disability. Priorities in the development of the resource-base include addressing neglected topics, standardizing recommendations, and operationalizing guidance for disability inclusion in both broader nutrition and disability-specific programming. Consideration must also be given to the human, financial and resource capacity of health systems to implement those recommendations and the visibility and accessibility of the recommendations globally.

Acknowledgments

Ali Murray

References

1. Levels and trends in child malnutrition. UNICEF / WHO / World Bank Group Joint Child Malnutrition Estimates. Key findings of the 2023 edition. 2023.

2. Zlotkin S, Dewey KG. Perspective: Putting the youngest among us into the nutrition “call for action” for food fortification strategies. Am J Clin Nutr. 2021 Oct;114(4):1257–60.

3. Dr Di Cesare M, Dr Ghosh S, Dr Osendarp S, Dr Mozaffarian D. A world free from malnutrition: An assessment of progress towards the global nutrition targets. 2021. (https://globalnutritionreport.org/50bff8#section-2-1 (Accessed 23/08/24, 11.45)). Report No.: Chapter 2: The Burden of Malnutrition.

4. WHO. Global Report on Health Equity for Persons with Disabilities. Geneva; 2022.

5. United Nations Children’s Fund. Seen, Counted, Included: Using data to shed light on the well-being of children with disabilities. New York; 2021.

6. Olusanya BO, Kancherla V, Shaheen A, Ogbo FA, Davis AC. Global and regional prevalence of disabilities among children and adolescents: Analysis of findings from global health databases. Front Public Health. 2022 Sep 23;10.

7. Olusanya BO, Boo NY, Nair MKC, Samms-Vaughan ME, Hadders-Algra M, Wright SM, et al. Accelerating progress on early childhood development for children under 5 years with disabilities by 2030. Lancet Glob Health. 2022 Mar;10(3):e438–44.

8. Olusanya BO, de Vries PJ, Olusanya BO, Gladstone M, Rukabyarwema JP, Kuper H, et al. Nurturing care for children with developmental disabilities: a moral imperative for sub-Saharan Africa. Lancet Child Adolesc Health. 2018 Nov;2(11):772–4.

9. Kerac M, Postels DG, Mallewa M, Alusine Jalloh A, Voskuijl WP, Groce N, et al. The Interaction of Malnutrition and Neurologic Disability in Africa. Semin Pediatr Neurol. 2014 Mar;21(1):42–9.

10. Nishioka S, Wakabayashi H. Interaction between malnutrition and physical disability in older adults: is there a malnutrition-disability cycle? Nutr Rev. 2023 Jan 10;81(2):191–205.

11. Liu R, Pi L, Leng F, Shen Q. Global disability-adjusted life years and deaths attributable to child and maternal malnutrition from 1990 to 2019. Front Public Health. 2024 Jan 18;12.

12. Rotenberg S, Chen S, Hunt X, Smythe T, Kuper H. Are children with disabilities more likely to be malnourished than children without disabilities? Evidence from the Multiple Indicator Cluster Surveys in 30 countries. BMJ Nutr Prev Health. 2024 Jun;7(1):38–44.

13. Hume-Nixon M, Kuper H. The association between malnutrition and childhood disability in low- and middle- income countries: systematic review and meta-analysis of observational studies. Tropical Medicine & International Health. 2018 Nov 10;23(11):1158–75.

14. Lelijveld N, Groce N, Patel S, Nnensa T, Chimwezi E, Gladstone M, et al. Long-term outcomes for children with disability and severe acute malnutrition in Malawi. BMJ Glob Health. 2020 Oct 7;5(10):e002613.

15. United Nations Children's Fund (UNICEF). UNICEF Conceptual Framework on Maternal and Child Nutrition. 2021 Nov.

16. Groce N, Challenger E, Berman-Bieler R, Farkas A, Yilmaz N, Schultink W, et al. Malnutrition and disability: unexplored opportunities for collaboration. Paediatr Int Child Health. 2014 Nov 13;34(4):308–14.

17. Sixty-first session of the General Assembly by resolution A/RES/61/106. Convention on the Rights of Persons with Disabilities. In: Human Rights Instrument. United Nations; 2006.

18. United Nations. Convention on the Rights of the Child. In: General Assembly resolution 44/25, Article 49. 1989.

19. Hannah Kuper, Phyllis Haydt. The Missing Billion: ACCESS TO HEALTH SERVICES FOR 1 BILLION PEOPLE WITH DISABILITIES. 2019 Jul.

20. Groce NE, Kerac M, Farkas A, Schultink W, Bieler RB. Inclusive nutrition for children and adults with disabilities. Lancet Glob Health. 2013 Oct;1(4):e180–1.

21. Klein A, Uyehara M, Cunningham A, Olomi M, Cashin K, Kirk CM. Nutritional care for children with feeding difficulties and disabilities: A scoping review. PLOS Global Public Health. 2023 Mar 17;3(3):e0001130.

22. USAID and Advancing Nutrition. https://www.advancingnutrition.org/resources/disability-resource-bank [Accessed 29/08/2024 10.57]. Feeding and Disability Resource Bank: A collection of resources to address feeding difficulties and disability inclusion in nutrition programs.

23. United Nations Children's Fund (UNICEF). Feeding and Disability Resource Bank [Internet]. 2024 [cited 2024 Jun 3]. Available from: https://knowledge.unicef.org/feeding-and-disability-resource-bank/landing-page

24. Saunders CH, Sierpe A, von Plessen C, Kennedy AM, Leviton LC, Bernstein SL, et al. Practical thematic analysis: a guide for multidisciplinary health services research teams engaging in qualitative analysis. BMJ. 2023 Jun 8;e074256.

25. Physiopedia. https://www.physio-pedia.com/Managing_Feeding_in_Children_with_Cerebral_Palsy. p. (n.d) Managing Feeding in Children with Cerebral Palsy.

26. SPOON. Resources [Internet]. [cited 2024 Jun 4]. p. (n.d). Available from: https://www.spoonfoundation.org/library/

27. SPOON. Introduction to Nutrition and Feeding for Children [Internet]. [cited 2024 Jun 4]. p. (n.d). Available from: https://www.spoonfoundation.org/intro-course/#:~:text=SPOON’s%20free%20online%20course%20Introduction,health%20app%2C%20Count%20Me%20In.

28. Thelma Tun Thein, Khin May Tun Chit. Nutrition Integration for the children with special needs. 2020.

29. Kaplan T, Kaui E, Watts B, Catt R, Miller R, DeLacey E, et al. Holt International’s Feeding and Positioning Manual: Guidelines for working with babies and children [Internet]. Holt International; 2019 [cited 2024 Jun 4]. Available from: https://www.holtinternational.org/nutrition-health/feeding-positioning-manual/

30. de Silva H, Asir M. Working with Infants with Feeding Difficulties. 1st ed. Multi-agency International Training and Support; 2018.

31. Adams M, Pettigrew J. Working with Children with Eating and Drinking Difficulties - A training Programme for healthcare specialists in low-resource settings. MAITs Multi Agency International Training and Support; 2017.

32. Global health Media. Our Videos: Care of Small Babies [Internet]. [cited 2024 Jun 4]. Available from: https://globalhealthmedia.org/topic/small-baby/

33. Yang HR. How to approach feeding difficulties in young children. Vol. 60, Korean Journal of Pediatrics. Korean Pediatric Society; 2017. p. 379–84.

34. Gladstone M, Mallewa M, Alusine Jalloh A, Voskuijl W, Postels D, Groce N, et al. Assessment of neurodisability and malnutrition in children in Africa. Semin Pediatr Neurol. 2014;21(1):50–7.

35. Health Resources and Services Administration UD of H and HS. The CDC Growth Charts for Children with Special health Care Needs [Internet]. [cited 2024 Jun 4]. Available from: https://depts.washington.edu/growth/cshcn/text/page1a.htm

36. Samson-Fang L, Bell KL. Assessment of growth and nutrition in children with cerebral palsy. Eur J Clin Nutr. 2013;67:S5–8.

37. Arts-Rodas DR, Benoit FRCPC D. Feeding problems in infancy and early childhood: Identification and management.

38. USAID Advancing Nutrition. Responsive Care and Early Learning Addendum Resources in English [Internet]. Arlington, VA: USAID Advancing Nutrition;

2023 [cited 2024 Jun 4]. Available from: https://www.advancingnutrition.org/resources/responsive-care-early-learning-addendum/english

39. Holt International. Child Nutrition Programme, Community Flipbook. 2022 Mar [cited 2024 Jun 4]; Available from: https://www.holtinternational.org/wp-content/uploads/2022/03/Flip-book-English-Final-3.1.22.pdf

40. Ubuntu. Ubuntu Manual: Working Together with Families of Children with Developmental Disabilities. 2021 [cited 2024 Jun 4]; Available from: https://www.ubuntu-hub.org/resources/working-together-with-families-and-children-with-developmental-disabilities/

41. Ubuntu. Baby Ubuntu. 2021 [cited 2024 Jun 4]; Available from: https://www.ubuntu-hub.org/resources/babyubuntu/

42. Ubuntu. Juntos. 2019 [cited 2024 Jun 4]; Available from: https://www.ubuntu-hub.org/resources/juntos/

43. Adams M, Staple-Ebanks C, Joshi A, Mercer E, Rangwala S, Lassman R. Working with Children with Developmental Disabilities and their Caregivers. London; 2023.

44. United Nations Children’s Fund (UNICEF) and the International Step by Step Association. Supporting families for nurturing care: Resource modules for home visitors. 2016 [cited 2024 Jun 4]; Available from: https://www.issa.nl/modules_home_visitors

45. Christian Blind Mission. CBM Cerebral Palsy Prevention Toolkit. 2012 [cited 2024 Jun 4]; Available from: https://www.cbm.org/get-involved/search-resources-publications/?tx_wwt3site_list%5B__referrer%5D%5B%40extension%5D=Wwt3Site&tx_wwt3site_list%5B__referrer%5D%5B%40controller%5D=Publication&tx_wwt3site_list%5B__referrer%5D%5B%40action%5D=list&tx_wwt3site_list%5B__referrer%5D%5Barguments%5D=YTowOnt97a70dc1eec8c27f56c58e2b900cef02d68011f1e&tx_wwt3site_list%5B__referrer%5D%5B%40request%5D=a%3A3%3A%7Bs%3A10%3A%22%40extension%22%3Bs%3A8%3A%22Wwt3Site%22%3Bs%3A11%3A%22%40controller%22%3Bs%3A11%3A%22Publication%22%3Bs%3A7%3A%22%40action%22%3Bs%3A4%3A%22list%22%3B%7D13f1a9698cd594b129675b8ce62c64286924033e&tx_wwt3site_list%5B__trustedProperties%5D=a%3A0%3A%7B%7D75e6f8c6217790a6d75e6c070ade464ccdde848d&tx_wwt3site_list%5Bfilter%5D%5Byear%5D=0&tx_wwt3site_list%5Bfilter%5D%5Bcategory%5D=68&tx_wwt3site_list%5Bfilter%5D%5Btype%5D=4#resultlist

46. United Nations Children’s Fund (UNICEF). Essential Actions on Disability-Inclusive Nutrition. 2022;

47. United Nations Children’s Fund (UNICEF). Inclusion of Children and Young People with Disabilities in Routine General Health Care: Practice Guide. 2022 Aug;

48. United Nations Children’s Fund (UNICEF). Improving Young Children’s Diets During the Complementary Feeding Period. UNICEF Programming Guidance. New York. 2020 Feb;

49. World Health Organization Regional Office for the Western Pacific. Disability inclusive Health Services Toolkit: A Resource for Health Facilities in the Western Pacific Region. Manila, Philippines. 2020;

50. World Health Organisation. Monitoring children’s development in primary care services: moving from a focus on child deficits to family-centred participatory support. Report of a virtual technical meeting, 9-10 June 2020. 2020;

51. World Health Organisation and United Nations Children’s Fund (UNICEF). Nurturing Care Handbook, Strategic Action 4: Monitor Progress. How to monitor populations, implementation and individual children’s development. Geneva [Internet]. 2022 [cited 2024 Jun 4]; Available from: https://nurturing-care.org/nurturing-care-handbook-monitor-progress/

52. United Nations Children’s Fund (UNICEF). Including Children with Disabilities in Humanitarian Action: Nutrition. 2018 Feb [cited 2024 Jun 4]; Available from: https://www.unicef.org/disabilities/inclusive-humanitarian-action-guidance

53. Fernald LCH, Prado E, Kariger P, Raikes A. A Toolkit for Measuring Early Childhood Development in Low- and Middle-Income Countries. Prepared for the Strategic Impact Evaluation Fund, the World Bank. International Bank for Reconstruction and Development / The World Bank [Internet]. 2017 [cited 2024 Jun 4];Washington DC. Available from: https://hdl.handle.net/10986/29000

54. Washington Group on Disability Statistics. The Washington Group Short Set on Functioning – Enhanced (WG-SS Enhanced). 2022 Oct;

55. The Washington Group and United Nations Child Fund (UNICEF). Module of Child Functioning: Questionnaires. 2016 [cited 2024 Jun 4]; Available from: https://data.unicef.org/resources/module-child-functioning/

56. University of Liverpool. Malawi Development Assessment Tool (MDAT). 2020.

57. Robins D, Fein D, Barton M. Modified Checklist for Autism in Toddlers, Revised, with Follow-Up (M-CHAT-R/F). 2009.

58. Arvedson J, Browne J, Delaney A, Feuling M, Fischer E, Gisel E, et al. Infant and Child Feeding Questionaire [Internet]. 2020 [cited 2024 Jun 4]. Available from: https://questionnaire.feedingmatters.org/

59. Sellers D, Mandy A, Pennington L, Hankins M, Morris C. Development and reliability of a system to classify the eating and drinking ability of people with cerebral palsy. Dev Med Child Neurol. 2014 Mar 18;56(3):245–51.

60. Cichero J, Lam P, Steele C, Hanson B, Chen J, Dantas R, et al. Development of international terminology and definitions for texture-modified foods and thickened fluids used in dysphagia management: The IDDSI Framework. Dysphagia. 2017;32:293–314.

61. WHO. WHO Guideline on the Prevention and Management of Wasting and Nutritional Oedema. 2023 Nov.

62. Sphere Association. The Sphere Handbook: Humanitarian Charter and Minimum Standards in Humanitarian Response. Fourth Edition. Geneva: www.spherestandards.org/handbook; 2018.

63. United Nations Children's Fund (UNICEF). UNICEF Disability Inclusion Policy and Strategy (DIPAS) 2022-2030. New York; 2022 Dec.

64. The Lancet Public Health. Disability—a neglected issue in public health. Lancet Public Health. 2021 Jun;6(6):e346.

65. Engl M, Binns P, Trehan I, Lelijveld N, Angood C, McGrath M, et al. Children living with disabilities are neglected in severe malnutrition protocols: a guideline review. Arch Dis Child. 2022 Jul;107(7):637–43.

66. Wallace AS, Ryman TK, Dietz V. Experiences Integrating Delivery of Maternal and Child Health Services With Childhood Immunization Programs: Systematic Review Update. Journal of Infectious Diseases. 2012 Mar 1;205(suppl 1):S6–19.

67. Howson CP, Cedergren B, Giugliani R, Huhtinen P, Padilla CD, Palubiak CS, et al. Universal newborn screening: A roadmap for action. Mol Genet Metab. 2018 Jul;124(3):177–83.

68. Adams M, Cardozo P, Cherquit J, Christopher S, Divan G, Gericke T, et al. Caring for Children with Developmental Disabilities. A Guide for Parents living in low-resource settings. Multi-Agency International Training and Support (MAITS). 2017;London(2).

69. Holt International. The Child Nutrition Program: Community Resource Manual. 2022 [cited 2024 Jun 4]; Available from: https://www.holtinternational.org/wp-content/uploads/2022/02/Holt-Community-Resource-Manual-2.24.22.pdf

70. Brouwers MC, Kho ME, Browman GP, Burgers JS, Cluzeau F, Feder G, et al. AGREE II: Advancing guidelines development, reporting and evaluation in healthcare. CMAJ; 2010.

Appendix of Supporting Information Captions

S1: Titles, Publisher and Date of Resources included in the Thematic Analysis with corresponding numbers for reference in the Report

S2: Table on Format, Languages and Disabilities included in Training Manuals and Programmes

S3: Key Informant Interview Topic Guide

Figure 1

Click here to access/download;Figure;Figure1.tif

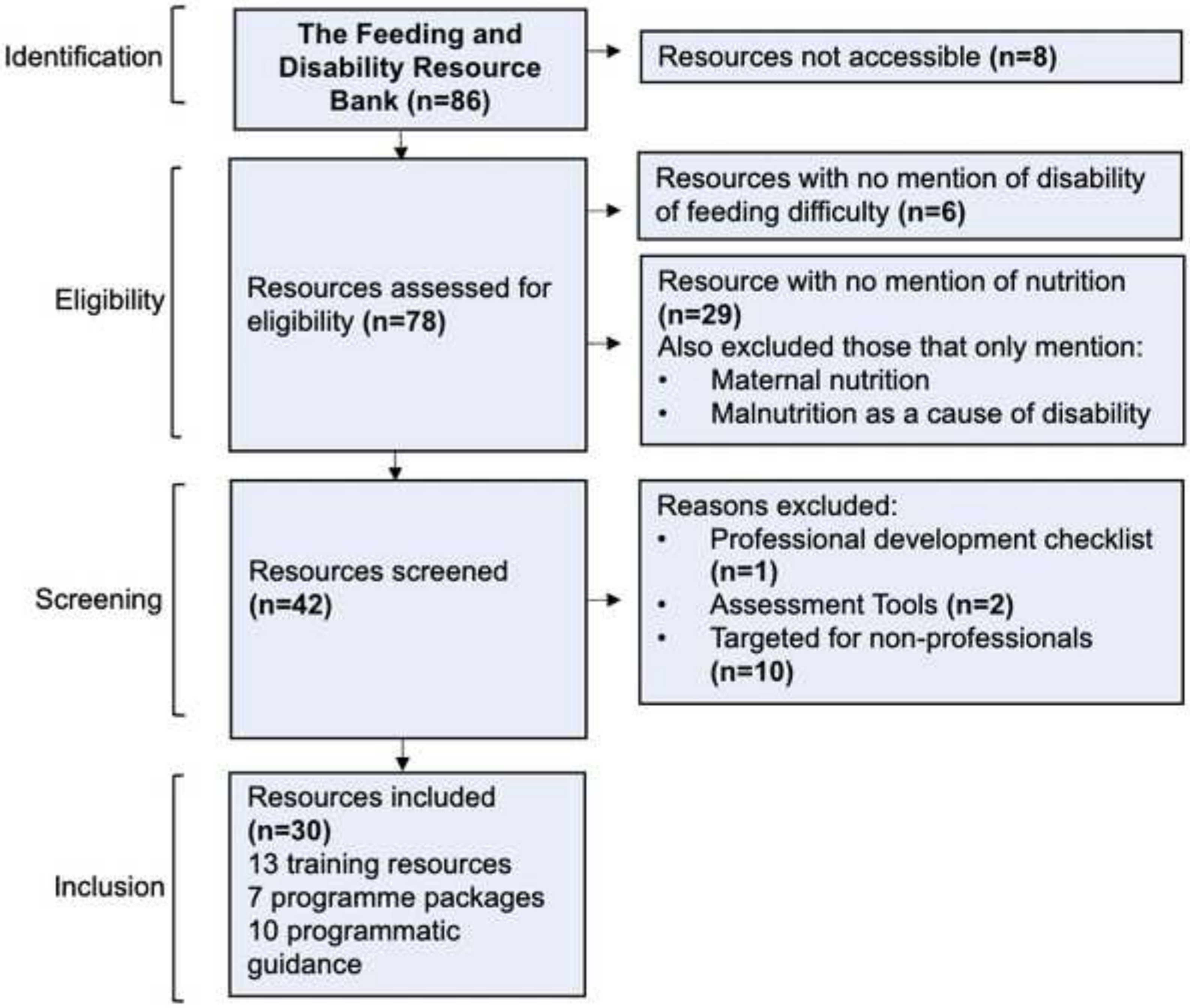

Figure 2

Click here to access/download;Figure;Figure2.tif

	Gaps	Standardise	Operationalise
Identification	• Screening tool to monitor development and disability in younger infants 0-2 years<br>• Recommendations for growth monitoring and malnutrition<br>• Formal method of feeding assessment	• Which development tools are most relevant to the nutrition context<br>• Caveats for inclusion criteria when anthropometric indicators are suitable	• How developmental monitoring, disability screening and identification of feeding difficulties should be implemented in nutrition programming
Management	• Guidance on nutrition quality in the context of restricted or modified diets<br>• Recommendations for managing hydration, reflux, vomiting, and more medically severe feeding difficulties	• Include and make more comprehensive recommendations for management into more program packages and programmatic guidance<br>• Inclusion of social, emotional and cognitive development and how this relates to feeding	• Include references to more comprehensive training manuals in guidelines
Integration	• Rights of a child with a disability, and approaches to the challenges of accessibility, stigma and discrimination in training materials (specific to nutrition context)<br>• Recommendations for vulnerable populations, such as those in institutional care, refugees, and migrants	• Guidance on social, financial and emotional support for families of children with disabilities<br>• Caveats to accommodate a child's need for longer-term care and support in nutrition programming	• Programmatic guidance and packages on how to implement an integrated and holistic clinic for children with disability<br>• Provide implementation guidance on how to identify and establish referral links between care

Figure 3 Click here to access/download;Figure;Figure3.tif

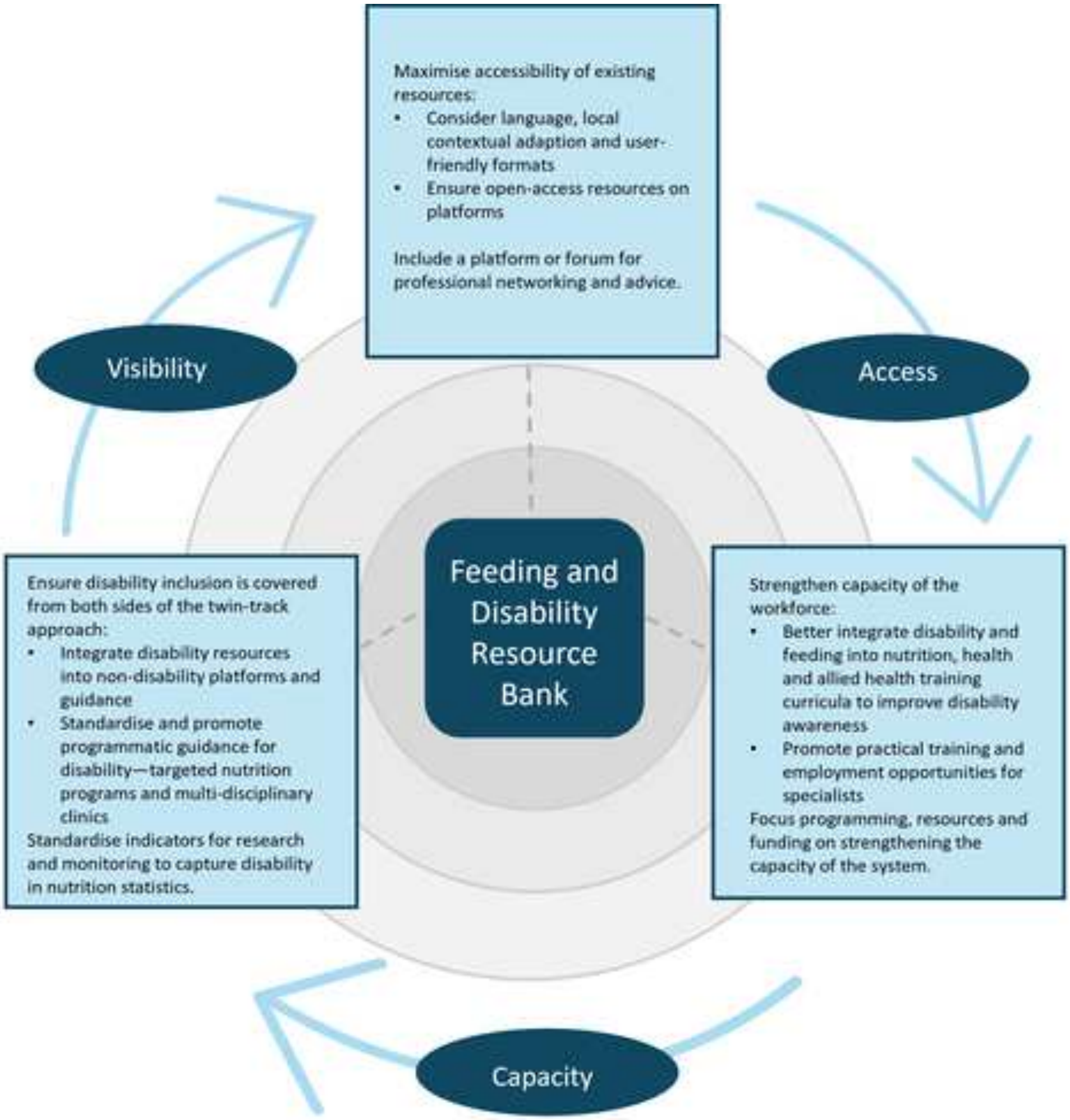

Click here to access/download
Supporting Information
Letter of Ethical Approval.pdf

Click here to access/download
Supporting Information
S1.docx

Click here to access/download
Supporting Information
S2.docx

Click here to access/download
Supporting Information
S3.docx
