# Supplementary material for: Children with disabilities in nutrition programmes: Thematic analysis of training and guidelines
Source: PLOS Glob Public Health. 2026 Apr 15;6(4):e0006188. doi: 10.1371/journal.pgph.0006188 (PMC13082666; doi:10.1371/journal.pgph.0006188)
Supplement: Attachment — Submitted filename: Response to Reviewers.docx [file pgph.0006188.s004.docx]

Dr. Jennifer Tucker, PhD
Staff Editor
PLOS Global Public Health

**Dear Dr. Tucker,**

**RE: Manuscript Submission – *Supporting Children with Disabilities in Nutrition Programmes: A Thematic Analysis of Training and Guidelines Key Informant Interviews***

Thank you for taking the time to review our manuscript and for the thoughtful feedback provided by you and the reviewers. We appreciate the opportunity to revise and strengthen our work.

We believe our paper is a strong fit for *PLOS Global Public Health* given:

1. Its relevance to global health equity and your journal’s mission to publish high-quality, transparent research.
2. Interest to your wide international readership of policy-makers, practitioners and researchers in global health: our work explores how to better include children with disabilities in nutrition services and is thus of direct benefit to those working in and planning front-line services for this vulnerable group.
3. Good track record of views and citations from a previous scoping review on nutrition/disability links which was published by your journal.^1^

Our key messages include that:

- Children with disabilities are frequently overlooked in nutrition and health programmes that could significantly improve their quality of life.
- More needs to be done to equip programmes and services to support vulnerable families and children with disabilities.
- Documents to improve nutrition and health services do exist and are highlighted by our work: using these would be an important step towards a fundamental human right, with inclusive programme design being essential for equitable health systems.

We have responded to each reviewer comment in detail and hope the revisions address the issues raised with a much stronger final version of our paper resulting.

Thank you again for your time and consideration. We look forward to the opportunity to move forward in the review process with *PLOS Global Public Health*.

Sincerely,

Katie Fulford MSc

**Response to Reviewers**

**Reviewer 1**

Thank you for your review, we are grateful for all your feedback. We appreciate your critique confirming that our statistical methods were performed appropriately and rigorously, and that our manuscript is technically sound. We hope that if this manuscript goes onto publication that our findings will be similarly trusted and appreciated by PLOS readers.

**Introduction**

**- Line 149-162: The aim and objectives of the study should be incorporated in the Introduction section.**

Response: Thank you for this feedback, we have updated our introduction section to integrate the aim and objectives.

**Material & methods:**
**- Lines 184-186: Justify why these materials were excluded - a comprehensive review to support a policy should include resources intended for all populations.**

Response: In the third paragraph of the Methods (line 169-179), we have further clarified our methods and why some resources were excluded based on our review criteria. Thank you for this suggestion.

**- Line 198: Provide specific data on the job specification of KI**

Response: We appreciate this suggestion; we have added a note in line 187-192 about the job specification of the KIs. Additional information is available in Table 1. Anonymity and confidentiality were a key considerations in this research, so only summary data is provided on the key informants. We are aware of and are fully transparent about potential limitations of our KI sample in the results section (line 716-721).

**- Line 210: Has this guide been pilot-tested for feasibility?**

Response: The guide was developed through extensive suggestions with co-authors who are experienced in this field. It was pilot tested with two initial practice interviews and modifications were agreed upon by co-authors. We found this to be feasible, acceptable, and effective at capturing relevant information. To make this clearer to future readers we have added a line in the Methods on page 12 (Lines 205-208).

**- Line 211: How many main and probing questions are in the guide?**

Response: Thank you for this question. We have expanded the explanation in lines 202-205 to include more about the topic guide. We had 11 main questions and 0-4 probing questions to support each. To help readers understand our current work, but also to help other researchers replicate our study / do similar other research, we include our interview guide is in the appendix of supporting information (S3). We appreciate your time reviewing the guide.

**- Line 229: How many team members were involved during the analysis? How do you ensure the validity of the analysis?**

Response: In our authorship statement on page 2, we specify each author's role in this research. KF led on the data extraction and analysis of the transcripts with support and supervision from senior author MK. Interpretation of the analysis and overall validity was ensured by review and contribution to the paper by all authors. We would prefer to keep the paper more focused and shorter since we believe that is helpful to readers, but If the journal would like this information added to the methods section, we would be happy to do so.

**Results:**
**- Line 250: Prior to this statement, provide characteristics of reviewed literature - origin country, intended for which type of disabilities, etc.**

Response: Thank you for this suggestion. The initial scoping review to identify the resources in the bank, which we used for our research, included a published paper in 2023 demonstrating the key characteristics of each resource found^[1]^. To build on this previous paper, we decided to focus on the content of each resource and have summarized some characteristic information about the resources in Tables 2, 4 and 5. We aimed to support and build on, rather than fully recreate, the characteristics provided in the earlier paper published by PLOS Global Public Health. Our flow chart summarizes how we used and categorized these documents from the previous review. We included a table in the appendix (S1) outlining characteristics of each document that may contribute to the identified barriers and facilitators to their use including the type of disabilities mentioned, but also formats available and languages translated.

**- Results for literature analysis and KI interview should be separated. The current arrangement of results is difficult to understand and lacks flow for easy readability.**

Response: We appreciate this point, and we have gone through and made edits to improve the readability in the results section “Recommendations for future development of resources”. We discussed this at length as an author team and in an earlier version we had the results of the literature analysis and key informant interviews separate but found it to be less clear than the integrated approach which summarized the key findings together. The aim of the interviews was to provide case examples and professional’s real world experience to show that the issues identified in the analysis are impacting current practice of care. We appreciate that different readers will have different preferences, so we hope the edits we have made improve readability and clarity to continue with this presentation.

**- The use of a heatmap is appropriate; however, the organization of the information is unclear.**

Response: Thanks for this comment, we appreciate this feedback. During writing, we experimented with numerous different variations to discover what works best to display this information. We found splitting the table by content area: identification, management and integration, was more reader-friendly than other ways of organizing. Using the resource title, author and date of publication in table 2- but using titles only in table 4 and 5, we believe gives readers more clarity when viewing the heat maps. We have however adjusted the format of the Heat Maps (Table 2, 4 and 5) to improve readability of the information. We envision journal formatting will further help the final published version: pop-up tables will address the space required for the full tables to be easily reviewed instead of embedded within the text.

**Discussion:**
**- Should start with a summary of findings.**

Response: Thank you for this suggestion, we have revised to combine more summary information in the beginning of the discussion section. (Page 42) We have made some edits and followed your suggestion.

**- Lack of elaboration on the key findings and why each key finding is essential in the construction of policy.**

Response: We appreciate this suggestion and have added some information to elaborate further on our key findings and why they are essential in support for policy development.

**- Incoherent from one paragraph to another.**

Response: In addressing the other comments we have made lots of edits to our work and trust that this also improves para to para coherence of our discussion section. We have reorganized and rephrased this whole section and trust that it is now clearer and better as a result. (Pages 42-43)

**Conclusion:**
**- Does not conclude the aim and objective of the study.**

Response: Thank you for raising this. To address this suggestion, we have added a line to summarize the aim and objectives of the studies and reorganized the conclusion for additional information. (Page 47) We trust that the sentences now map more directly to the study objectives.

**Acknowledgement:**
**- Incomplete information**

Response: We have further expanded our acknowledgement section to provide complete information.

1. Klein A, Uyehara M, Cunningham A, Olomi M, Cashin K, Kirk CM (2023) Nutritional care for children with feeding difficulties and disabilities: A scoping review. PLOS Glob Public Health 3(3): e0001130. <https://doi.org/10.1371/journal.pgph.0001130>
