# Supplementary material for: Children with disabilities in nutrition programmes: Thematic analysis of training and guidelines
Source: PLOS Glob Public Health. 2026 Apr 15;6(4):e0006188. doi: 10.1371/journal.pgph.0006188 (PMC13082666; doi:10.1371/journal.pgph.0006188)
Supplement: Attachment — Submitted filename: Response to Editor and Reviewer (1).docx [file pgph.0006188.s005.docx]

Prof Razak M Gyasi, PhD, PD
Academic Editor
PLOS Global Public Health

Dear Professor Razak M Gyasi,

**RE: Revised Submission – “Children with Disabilities in Nutrition Programmes: A Thematic Analysis of Training and Guidelines”**

Thank you very much for the insightful feedback from you and the reviewers. We appreciate the time and expertise that went into your review and we welcome the opportunity to improve our work.

We remain confident that our study is a strong fit for *PLOS Global Public Health*. The research addresses a significant and under-examined issue — the frequent exclusion of children with disabilities from nutrition and health programmes — which has profound implications for global health equity. By exploring how training and guidelines do (or do not) support inclusion, our manuscript advances understanding of a critical policy and implementation gap. Our work builds on and complements a prior scoping review (also published in your journal) that documented this gap.

Below, we provide detailed, point-by-point responses to each comment (with changes highlighted in yellow in the revised manuscript). We hope that our revisions meet the expectations of you and the reviewer. We remain very willing to make additional revisions if desired. We have also included a visual abstract of this research to enhance engagement across diverse audiences. If the journal would like to include, we can update to include the doi or link to the journal to ease sharing

Thank you again for your consideration and for your constructive guidance. We look forward to the possibility of continuing through the review process with *PLOS Global Public Health*.

Sincerely,

Katie Fulford, MSc (on behalf of all co-authors)

We appreciate the editor and reviewer’s feedback. Thank you to reviewer 1 for the feedback that we have addressed all their comments, and they find our manuscript suitable for publication. Specifically, we are happy that they found our manuscript technically sound, our statistical analysis appropriate and well done. Your time has been much appreciated in support of improving our manuscript. Below we address all the final comments to the editor and are happy to update or clarify anything further.

1. Please review your reference list to ensure that it is complete and correct... Any changes to the reference list should be mentioned in the rebuttal letter that accompanies your revised manuscript.

Response: Thank you for this feedback as it was a great point to raise, and we have consequently reviewed all citations.

All the peer-reviewed published research articles in the paper are correct and still available.

The references for the resources - guidelines, manuals, reports, web pages were originally accessed from the USAID Feeding and Disability Resource Bank. Unfortunately, this page no longer exists and the resource bank has been transitioned to UNICEF. As discussed in the Methods section, most of the resources are now accessible from this new UNICEF platform, and therefore I have updated the references to reflect this/removed any web address that no-longer exist.

| Number | Change made | New Reference |
| --- | --- | --- |
| Ref 25 | Link updated | Physiopedia. https://www.physio-pedia.com/Feeding_the_Child_with_Cerebral_Palsy (n.d) Managing Feeding in Children with Cerebral Palsy. [cited 2024 Jun 4 |
| Ref 28 | No longer available online, weblink removed. | Thelma Tun Thein, Khin May Tun Chit. Nutrition Integration for the children with special needs. LEARN. 2020. |
| Ref 37 | Updated reference with missing information | Arts-Rodas DR, Benoit FRCPC D. Feeding problems in infancy and early childhood: Identification and management. Paediatr Child Health. 1998 Jan;3(1):21-7. |
| Ref 39 | Link updated | Holt International. Child Nutrition Programme, Community Flipbook. 2022 March [cited 2024 Jun 4]. Available from: https://www.holtinternational.org/research/ |
| Ref 44 | Training programme no longer available as new updated 2025 version published. Weblink removed. | United Nations Children’s Fund (UNICEF) and the International Step by Step Association. Supporting families for nurturing care: Resource modules for home visitors. 2016 [cited 2024 Jun 4] |
| Ref 45 | No longer available online, weblink removed. | Christian Blind Mission. CBM Cerebral Palsy Prevention Toolkit. 2012 [cited 2024 Jun 4] |
| Ref 53 | Link updated | Fernald LCH, Prado E, Kariger P, Raikes A. A Toolkit for Measuring Early Childhood Development in Low- and Middle-Income Countries. Prepared for the Strategic Impact Evaluation Fund, the World Bank. International Bank for Reconstruction and Development / The World Bank [Internet]. 2017 ;Washington DC. [cited 2024 Jun 4] Available from: https://openknowledge.worldbank.org/entities/publication/deb106bb-7361-55c3-9c3d-edb33986a1e6 |
| Ref 69 | Link updated | Holt International. The Child Nutrition Program: Community Resource Manual. 2022 [cited 2024 Jun 4]; Available from: https://www.holtinternational.org/research/ |

2. Please, provide the context of the study - the country or region where the nutrition intervention program took place in the title. Also, the second part of the title ("thematic analysis of training and guidelines key informant interviews") appears incomplete as if something is missing. Please revise this for clarity.

Response: Thank you for this feedback, we have reviewed and updated, as indicated by yellow highlight on page 11, 12 and 14.

For further clarification, data collection and analysis were conducted from the UK, however the context of the study was global. The 30 resources were retrieved from a global resource bank, collectively published from 11 different countries. The resources were analysed in English, but various versions of the documents were available in 36 different languages and the implementation of the resources is largely focused in low- and middle- income countries. Interviews were held virtually with participants from 8 different countries, with collective professional experience working across Asia and the Pacific, Middle East and North Africa, Sub-Saharan Africa, Europe and North America.

3. Data should also go with a plural verb. Revise and replace "data was or is" with "data were or are" throughout the manuscript.

Response: Thank you for this feedback, we have reviewed the manuscript’s tensing and updated, as indicated by yellow highlight.

4. Please, provide clear mechanistic pathways for all important findings at the discussion section of the paper.

Response: Thank you for this excellent suggestion feedback, we have added clearer information about the mechanistic pathways in the results section, and then summarised these at the start of the discussion. We hope this helps to identify our key findings and the implications of these on nutritional outcomes for children with disabilities. This has helped us to stress the key areas to improve on in future resource development. These changes have been highlighted in yellow on page 34 (Results: recommendations for future development of resources), and page 42 (Discussion).

5. Please do your best to clearly contextualize your findings - your discussion should be amiable to both localised but should highlight international relevance in order to globalize your study for a wider audience.

Response: Thank you for this feedback, we have reviewed and updated, as indicated by yellow highlight in the discussion. This analysis was done on a global programme and policy level, and therefore comments are unable to be made at a localised level. However, as described in the discussion, reviews of national guidelines have found similar results. Hopefully, by clarifying the context of the study earlier in the paper, as in review point 1, we have demonstrated the international relevance of the findings more clearly.

6. Explain how the study limitations did not undermine the veracity of the findings.

Response: Thank you for this feedback, we have reviewed and updated throughout the limitations section, as indicated by yellow highlight.
